# Functional Roles of *FgLaeA* in Controlling Secondary Metabolism, Sexual Development, and Virulence in *Fusarium graminearum*

**DOI:** 10.1371/journal.pone.0068441

**Published:** 2013-07-16

**Authors:** Hee-Kyoung Kim, Seunghoon Lee, Seong-Mi Jo, Susan P. McCormick, Robert A. E. Butchko, Robert H. Proctor, Sung-Hwan Yun

**Affiliations:** 1 Department of Medical Biotechnology, Soonchunhyang University, Asan, Republic of Korea; 2 Bacterial Foodborne Pathogens and Mycology Research Unit, National Center for Agricultural Utilization Research, Agricultural Research Service, United States Department of Agriculture, Peoria, Illinois, United States of America; University of Wisconsin - Madison, United States of America

## Abstract

*Fusarium graminearum*, the causal agent of Fusarium head blight in cereal crops, produces mycotoxins such as trichothecenes and zearalenone in infected plants. Here, we focused on the function of *FgLaeA* in *F. graminearum*, a homolog of *Aspergillus nidulans LaeA* encoding the global regulator for both secondary metabolism and sexual development. Prior to gene analysis, we constructed a novel luciferase reporter system consisting of a transgenic *F. graminearum* strain expressing a firefly luciferase gene under control of the promoter for either *TRI6* or *ZEB2* controlling the biosynthesis of these mycotoxins. Targeted deletion of *FgLaeA* led to a dramatic reduction of luminescence in reporter strains, indicating that *FgLaeA* controls the expression of these transcription factors in *F. graminearum*; reduced toxin accumulation was further confirmed by GC-MS analysis. Overexpression of *FgLaeA* caused the increased production of trichothecenes and additional metabolites. RNA seq-analysis revealed that gene member(s) belonging to ∼70% of total tentative gene clusters, which were previously proposed, were differentially expressed in the Δ*FgLaeA* strain. In addition, Δ*FgLaeA* strains exhibited an earlier induction of sexual fruiting body (perithecia) formation and drastically reduced disease symptoms in wheat, indicating that *FgLaeA* seems to negatively control perithecial induction, but positively control virulence toward the host plant. *FgLaeA* was constitutively expressed under both mycotoxin production and sexual development conditions. Overexpression of a *GFP-FgLaeA* fusion construct in the Δ*FgLaeA* strain restored all phenotypic changes to wild-type levels and led to constitutive expression of GFP in both nuclei and cytoplasm at different developmental stages. A split luciferase assay demonstrated that FgLaeA was able to interact with FgVeA, a homolog of *A. nidulans* veA. Taken together, these results demonstrate that FgLaeA, a member of putative FgVeA complex, controls secondary metabolism, sexual development, and virulence in *F. graminearum*, although the specific regulation pattern differs from that of LaeA in *A. nidulans*.

## Introduction

*Fusarium graminearum* (teleomorph: *Gibberella zeae*), a homothallic ascomycetous fungus, is an important pathogen of major cereal plants that causes diseases such as head blight (scab), stalk rot, and ear rot [1]–[3]. Previously classified as a single panmictic species, the *F. graminearum* species complex consists of over 16 phylogenetically distinct species or lineages found throughout the world [4]–[9]. The ability of *F. graminearum* to produce sexual progeny (ascospores) on overwintering cereal debris is essential for completion of the recurrent cycle of the plant diseases [10]. In addition to direct yield losses, *F. graminearum* produces mycotoxins such as trichothecenes and zearalenone in host plants, threatening human and animal health [2]. Trichothecenes, potent inhibitors of eukaryotic protein biosynthesis, are associated with feed refusal, vomiting, diarrhea, dermatitis, and hemorrhages in farm animals [2], [11]. Trichothecenes also appear to contribute to the virulence of *F. graminearum* on host plants [12]. Zearalenone causes estrogenic disorders in laboratory rats, mice, and farm-raised swine [2].

Genes involved in the biosynthesis of mycotoxins and other secondary metabolites (e.g., those encoding metabolic enzymes and transporters) usually reside close to one another on a chromosome, forming a gene cluster. Expression of these gene clusters is controlled at multiple levels. A pathway-specific transcription factor located within a gene cluster can regulate the expression of other members of the same cluster (e.g., *TRI6* and *ZEB2* for the regulations of trichothecenes and zearalenone biosynthesis, respectively, in *F. graminearum*) [13], [14]. Expression of these pathway-specific transcription factors is affected by environmental conditions such as ambient pH and availability of nitrogen, which are in turn regulated by other types of transcription factors, often located outside of the gene cluster (e.g., AreA and/or PacC regulate the gene clusters for gibberellic acid, fumonisin, and trichothecenes) [15]–[19].

In addition to specific transcription factors, the discovery of global regulators provides new insight into the regulatory hierarchy for fungal secondary metabolites. These master regulators operate in a relatively nonspecific manner by acting at the level of chromatin remodeling. One of the most intensively studied global regulators is LaeA, a nuclear protein containing a methyltransferase domain. It was first identified as a positive regulator of various secondary metabolites in *Aspergillus nidulans* [20]. It has been shown to modulate the function of the so-called velvet (VeA) complex consisting of at least 10 different proteins including VeA, VeA-like, VelB, and VosA. This complex coordinates both secondary metabolism and morphological development in response to light [21], [22]. Under dark conditions, LaeA interacts with the VeA–VelB heterodimeric complex in the nucleus, forming the heterotrimeric VeA complex (VelB–VeA–LaeA), which is responsible for activating secondary metabolism and regulating asexual/sexual development [21], [22]. Additionally, LaeA has been shown to control both the protein levels of other VeA members (VosA, VelB, and VeA), as well as physical interactions between them, thereby indirectly regulating fungal development [22].

The functional roles of LaeA have been demonstrated in several fungal species including *Aspergillus* and *Penicillium* (Eurotiomycetes) [20], [23]–[26], *Cochliobolus heterostrophus* (Dothideomycetes) [27], and *F. fujikuroi* [28], *F. verticillioides* [29], *F. oxysporum* [30], and *Trichoderma reesei* (Sordariomycetes) [31], [32]. In *F. graminearum*, homologs of three VeA complex members have been identified, two of which, FgVeA and FgVelB, have been shown to regulate mycotoxin production and fungal development, similar to those in *Aspergillus* spp. [33]–[35]. However, function of the *F. graminearum LaeA* homolog (designated *FgLaeA*) and the existence of the nuclear heterotrimeric FgVeA complex FgVelB–FgVeA–FgVelB have yet to be established.

In this study, we provide a detailed functional characterization of *FgLaeA* in *F. graminearum* using newly developed firefly luciferase reporter systems for toxin production and protein–protein interaction, respectively, and other strategies. Targeted gene deletion, complementation, and overexpression demonstrated that *FgLaeA* functions as a positive regulator in the production of various mycotoxins, asexual development (only in the dark), and virulence on host plant as well as in gene expression for the corresponding secondary metabolites, while acting as a negative regulator in sexual development (albeit in a light-independent manner). In addition, *in vivo* protein interaction between FgVeA and FgLaeA was clearly demonstrated. In contrast to other fungal LaeA homologs, the FgLaeA protein was constitutively localized in both the cytoplasm and nucleus under both dark and light conditions.

## Results

### Targeted Deletion, Complementation, and Overexpression of *FgLaeA*

**Figure 1:**
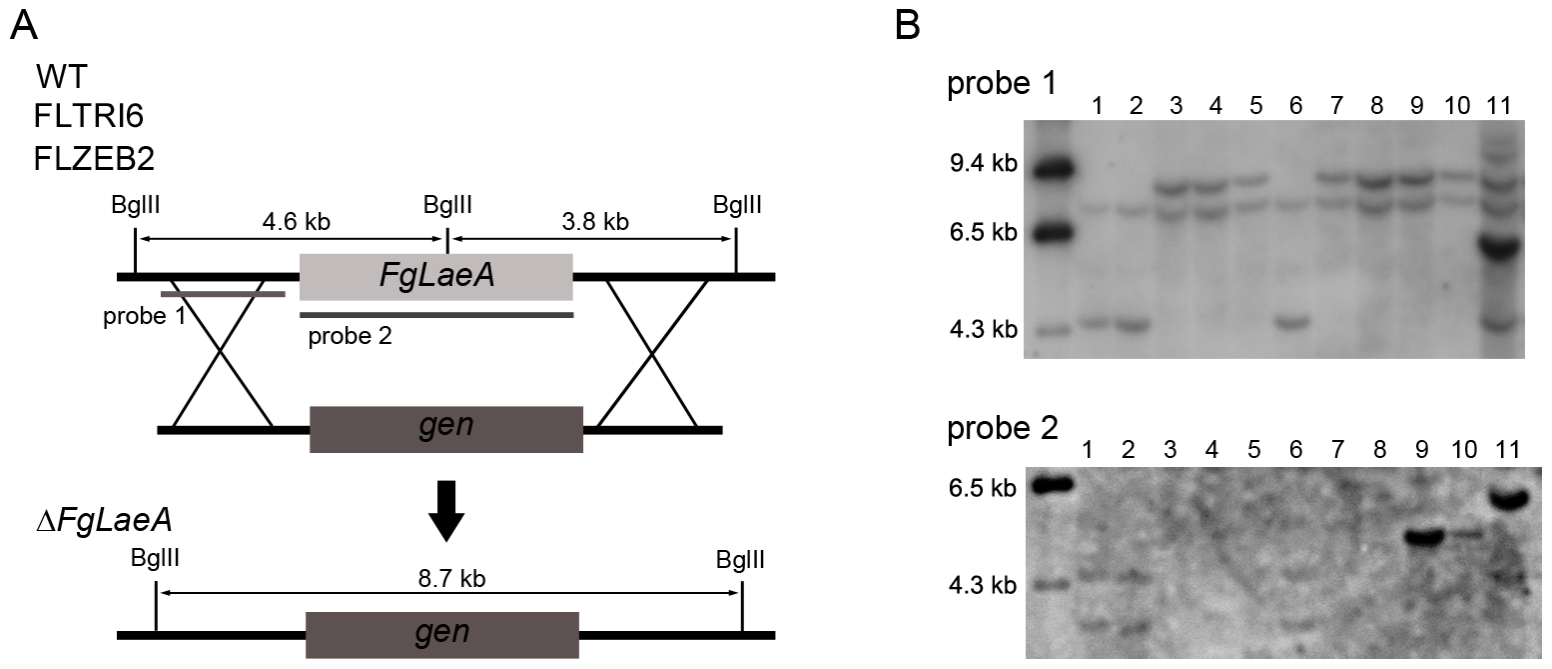
Targeted deletion of *FgLaeA* from the genome of the *F. graminearum* Z3643 (WT), FLTRI6, or FLZEB2 strains. (A) Deletion scheme. (B) *Bgl*II-digested genomic DNA gel blot hybridized with probe 1 (upper panel) and probe 2 (lower), respectively; positions are indicated in A. Lanes 1, 2, and 6: the Z3643, FLTRI6, and FLZEB2 strains; lanes 3 and 4: the *FgLaeA*-deletion strains derived from Z3643 (designated Δ*FgLaeA*); lanes 5 and 7: the *FgLaeA*-deletion strains derived from FLTRI6 (FLTRI6Δ*FgLaeA*); lane 7: the FLZEB2Δ*FgLaeA* strain; lanes 9 and 10: the *FgLaeA* add-back strains derived from the Δ*FgLaeA* strain; lane 11: the *FgLaeA*-overexpression strain derived from the Δ*FgLaeA* strain. DNA size markers are indicated on the left side of the gel. Note that a ∼7.5-kb nonspecific hybridizing band was observed in all of the strains examined when hybridized with probe 1.

A genome-wide search for homologs of the functionally characterized fungal *LaeA-*like genes, such as *A. nidulans LaeA* (GenBank accession no. AAQ95166.1) and *F. fujikuroi FfLae1* (FN548141), identified two open reading frames (ORFs) as possible LaeA homologs based on sequence homology, both carrying a conserved methyltransferase domain, in *F. graminearum*. The first ORF (annotated as FGSG_00657.3 in the *F. graminearum* genome database), encodes for 317 amino acids, interrupted by 6 introns, which was confirmed by reverse transcription-polymerase chain reaction (RT-PCR), but different from the previous annotation in the *F. graminearum* database. The intron positions are as follows: 25–87, 191–243, 393–445, 503–552, 703–749, and 1176–1222 (nucleotide numbering starts with 1 at the first nucleotide of the *FgLaeA* ORF as annotated in the genome database). Its coding region showed the highest similarity to FfLae1 (with 70% identity over 282 residues), but a lower similarity to LaeA (27.8% over 180 residues). We designated this ORF *FgLaeA*, as previously described [33], due to its highest sequence homology to *FfLae1* from a closely related *Fusarium* species. The second ORF (FGSG_07660, previously designated *FgVIP1* [33]) shared 35.8% identity to LaeA, and 30.6% identity to FfLae1 over 296 residues. Targeted deletion of both genes was performed in WT strain Z3643, resulting in the Δ*FgLaeA* and Δ*FgVIP1* strains, respectively. Integration of gene deletion constructs into the fungal genome via double crossover was verified by Southern blot (Fig. 1, data not shown for Δ*FgVIP1*). Because the Δ*FgVIP1* strains showed no dramatic changes in major fungal traits, further gene deletions using the FLTRI6 and FLZEB2 strains (the luciferase reporter strains for biosynthesis of trichothecenes and zearalenone, respectively; for details, see the subheading “Development of a firefly luciferase reporter system for mycotoxin production” in the result section) were done for only *FgLaeA*, generating the FLTRI6Δ*FgLaeA* and FLZEB2Δ*FgLaeA* strains, respectively (Fig. 1). Genetic complementation of a Δ*FgLaeA* strain derived from Z3643 was achieved by introducing an intact copy of *FgLaeA* (including ∼1 kb of its native promoter region) into the genome of the Δ*FgLaeA* strain, generating the so-called add-back strains (Fig. 1). For generating transgenic *F. graminearum* strains overexpressing *FgLaeA*, we complemented the Δ*FgLaeA* strain with an intact copy of the *FgLaeA* ORF fused to a green fluorescence protein gene (*GFP*) under control of a strong promoter from the *Cryphonectria parasitica Crp* gene [36], generating the OE::*GFP*-*FgLaeA* (OE) strain.

### Hyphal Growth, Pigmentation, Conidiation, and Sexual Development in the Δ*FgLaeA* and OE::*FgLaeA* Strains

**Figure 2:**
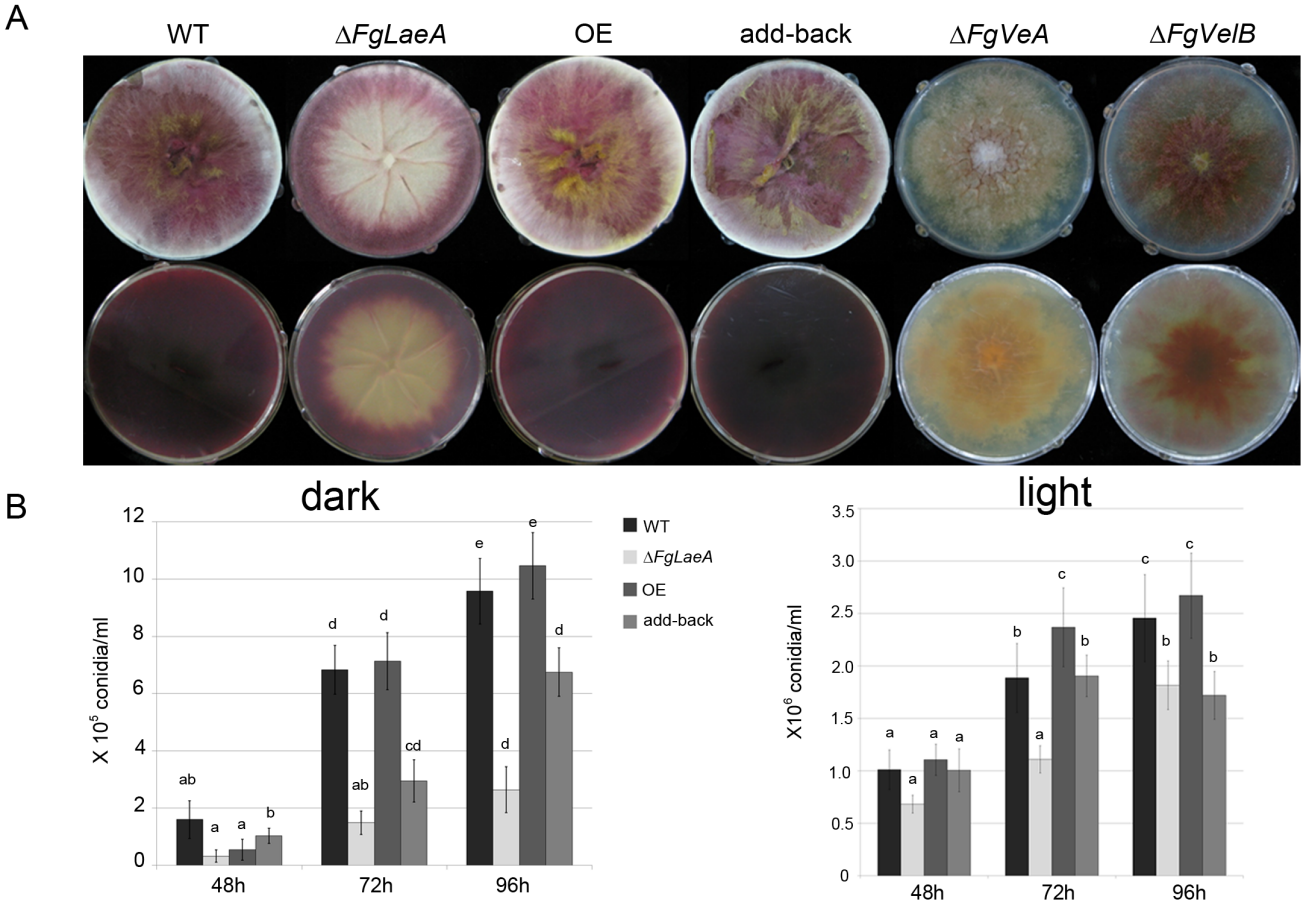
Hyphal growth, pigmentation, and conidiation. (A) Colony morphology of the WT (Z3643), Δ*FgLaeA*, OE (*FgLaeA*-overexpression), add-back, Δ*FgVeA* [32], and Δ*FgVelB* [33] strains on CM plates. Upper and lower panels show the morphology in surface and undersurface of plates, respectively. (B) The number of conidia formed in CMC liquid medium under dark and light conditions. The *y*-axis represents the number of conidia (×10^5^ or ×10^6^/mL). Data shown are the mean values obtained from three independent samples. Statistical analysis was performed with ANOVA and Duncan’s multiple range test. The same letter above bars represents no significant difference.

Compared to wild-type (WT) progenitors Z3643, FLTRI6, and FLZEB2, all Δ*FgLaeA* strains examined exhibited a slight reduction in radial growth, but a significant reduction in red hyphal pigmentation on potato dextrose agar (PDA; Difco Laboratories, Detroit, MI, USA), CM [3], and carrot agar (Fig. 2). However, this reduction was relatively modest compared with the phenotypes caused by the deletion of *FgVeA* [33] or *FgVelB* [34], which showed fewer aerial hyphae with reduced or abolished pigmentation (Fig. 2). The reduced pigmentation phenotype caused by Δ*FgLaeA* was less robust than a typical albino mutant of *F. graminearum* (e.g., that generated by a deletion of *PKS12*, the gene responsible for biosynthesis of the red pigment aurofusarin [37]). Aurofusarin production was still observed in young marginal hyphae of mutant colonies, but older regions of the colony were albino, resulting in ring-shaped pigmentation (Fig. 2). This pigmentation pattern was also seen in the marginal regions between different growing colonies (Fig. S1). Fewer aerial mycelia, with lower hydrophobicity were observed only on the albino region in the Δ*FgLaeA* strains; however, the formation of thick aerial hyphae was retained in the pigmented margin areas, similar to those from WT strains. This reduced pigmentation phenotype was restored to WT levels in both the *FgLaeA-*add-back and OE strains (Fig. 2). However, this phenotype is dependent upon growth conditions, as the same Δ*FgLaeA* strain was completely albino when grown in complete liquid medium (Fig. S1).

Conidiation of WT strains grown in carboxymethyl cellulose (CMC) liquid medium was strongly favored (∼10–15-fold increase) under 24-h light condition compared to those maintained in the dark. Δ*FgLaeA* strains showed no dramatic changes under light condition, while showed reduced levels of conidiation under dark condition (Fig. 2). No significant changes were detected among the WT, OE, and add-back strains (Fig. 2).

**Figure 3:**
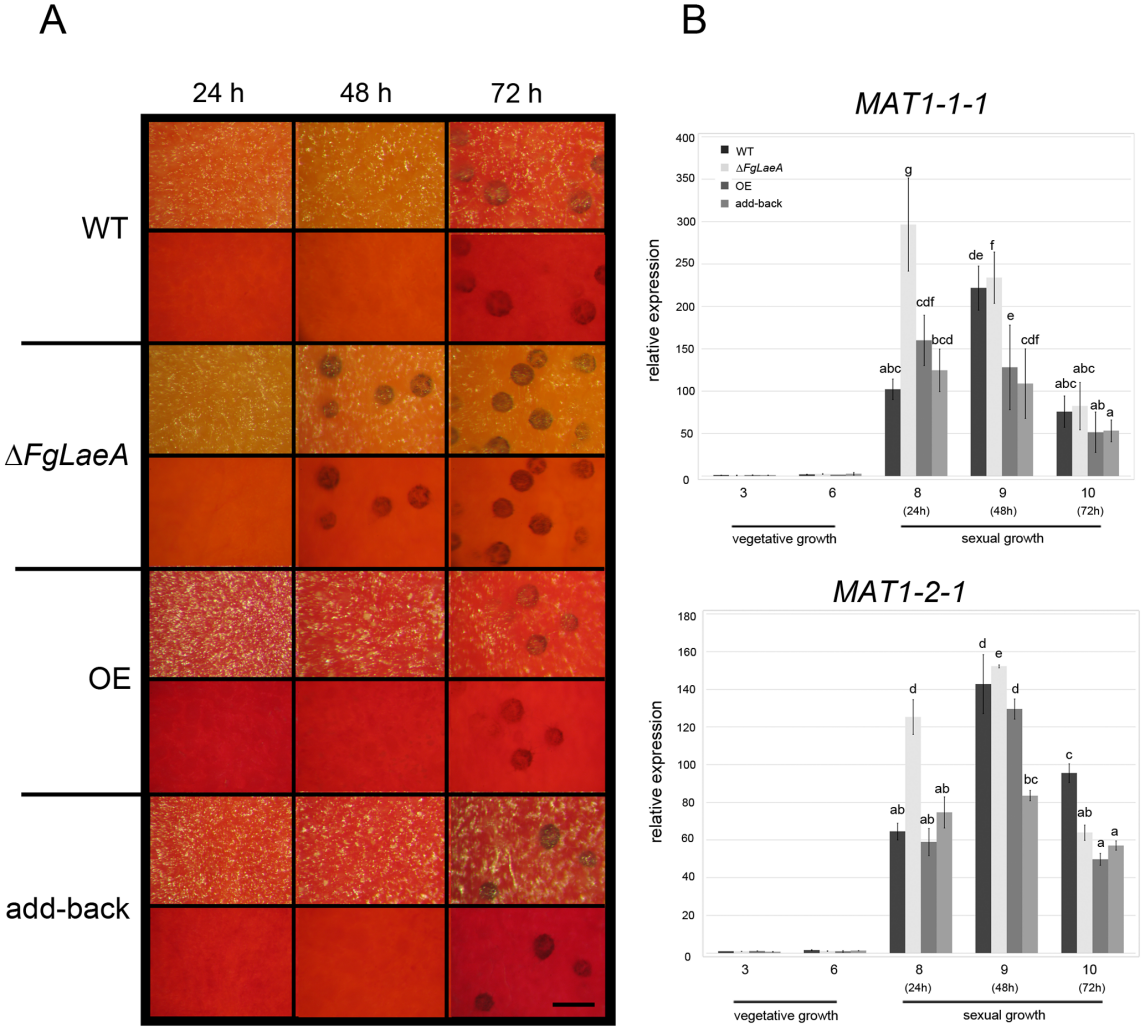
Protoperithecia formation and the expression of *MAT* genes. (A) Protoperithecia formation on carrot agar in the WT (Z3643), Δ*FgLaeA*, OE (*FgLaeA*-overexpression), and add-back strains. Time since perithecia induction (i.e., the removal of aerial mycelia, which had previously grown for 7 days in the dark) is indicated above the pictures. Upper and lower panels for each strain show the surface and undersurface, respectively, of carrot agar plates. The size bar indicates 200 µm. (B) Relative transcript levels for two *MAT* genes (*x*-axis) accumulated in the fungal strains shown in (A) at each of the growth time points (days, *y*-axis) for total RNA extraction. 3 and 6: days 3 and 6 under vegetative growth, respectively; 8, 9, and 10: days 8, 9, and 10 following perithecial induction, respectively (*i.e.* 24 h, 48 h, and 72 h after perithecia induction, respectively). *GzRPS16* (FGSG_09438.3) was used as an endogenous control for data normalization [52]. *MAT1-1-1* and *MAT1-2-1* transcript levels from a 3-day-old vegetative sample of a *F. graminearum* WT strain were used as references. Statistical analysis was performed with ANOVA and Duncan’s multiple range test. The same letter above bars represents no significant difference.

Sexual development (self-fertility) of Δ*FgLaeA* strains was determined based on perithecia production on carrot agar. The WT Z3643 strain began to produce protoperithecia approximately 72 h after sexual induction on carrot agar under both 24-h light and 12-h dark/light cycle conditions (Fig. 3). After an additional 3- or 4-day incubation, mature perithecia containing asci, each with eight ascospores formed. In contrast, five independent experiments confirmed that all Δ*FgLaeA* strains derived from the Z3643, FLTRI6, and FLZEB2 strains began producing protoperithecia approximately 24 h earlier than the WT strain under the same culture conditions (Fig. 3A). However, neither the WT nor Δ*FgLaeA* strains produced protoperithecia on carrot agar under 24-h dark conditions (data not shown). This early induction of protoperithecia formation in the Δ*FgLaeA* strains was restored to WT levels in both the add-back and OE strains (Fig. 3A). When a conidial suspension of the Δ*FgLaeA* strain was spermatized for the self-sterile Δ*MAT1-2* strain, fertile perithecia successfully formed on carrot agar, indicating that the Δ*FgLaeA* strain has no defect in male fertility (data not shown).

### Mycotoxin Production and Virulence of the Δ*FgLaeA* and OE::*GFP-FgLaeA* Strains

**Figure 4:**
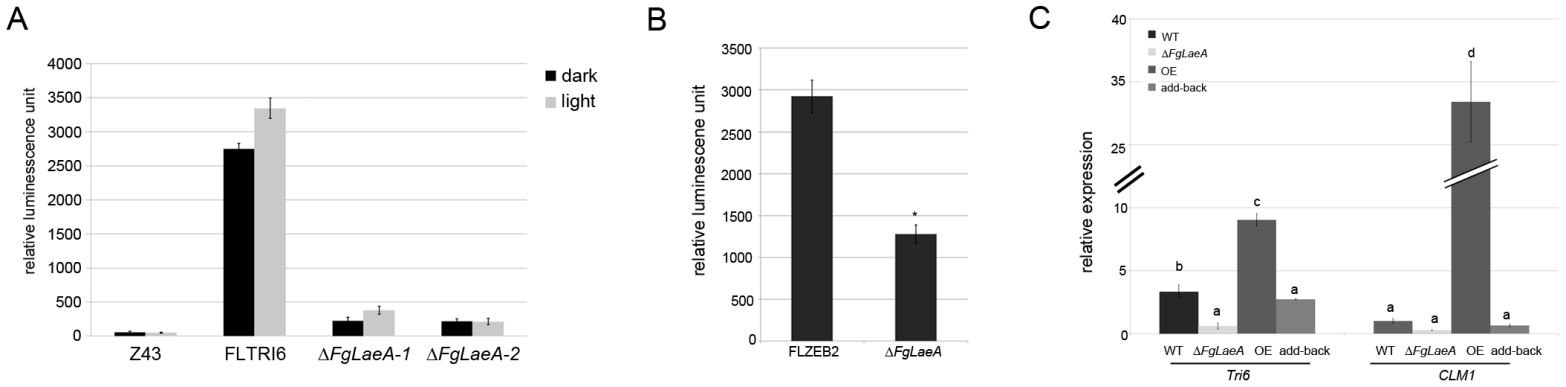
Mycotoxin production determined by luminescence signals and gene transcript levels. (A) Luminescence signals in the cell lysates from the Z3643 (Z43), FLTRI6, and two independent FLTRI6Δ*FgLaeA* (Δ*FgLaeA-1* and Δ*FgLaeA-2*) strains grown in AG liquid medium for 6 days. (B) Luminescence signals in cell lysates from the FLZEB2 and FLZEB2Δ*FgLaeA* (Δ*FgLaeA*) strains grown in SG liquid medium for 8 days. (C) Relative transcript levels for secondary metabolites accumulated in the WT, Δ*FgLaeA* (derived from Z3643), *FgLaeA*-overexpression (OE), and add-back strains grown in AG liquid medium for 6 days, as determined by qRT-PCR. The amount of *CLM1* transcript in the WT strain was used as a reference.//: discontinuity. Statistical analysis was performed with ANOVA and Duncan’s multiple range test. The same letter above bars represents no significant difference.

**Figure 5:**
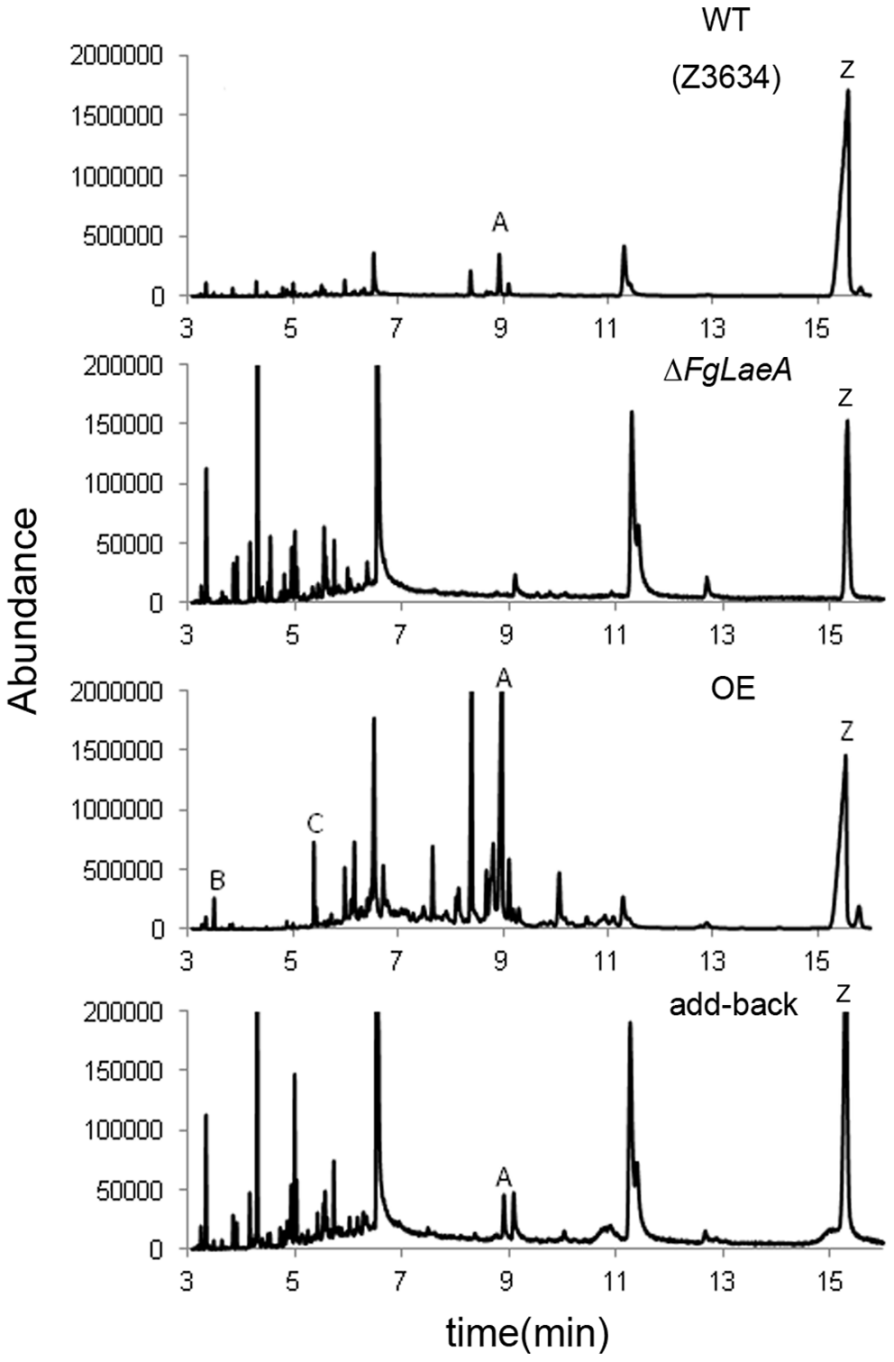
Reconstructed ion chromatogram of fungal rice culture extracts. WT (Z3643), the wild-type Z3643 strain; Δ*FgLaeA,* a Z3643Δ*FgLaeA* strain derived from Z3643; OE, a *FgLaeA-*overexpression strain derived from the Z3643Δ*FgLaeA* strain; add-back, a *FgLaeA*-add-back strain derived from the Z3643Δ*FgLaeA* strain. A, 15ADON; B, butenolide; C, culmorin; Z, zearalenone.

The effect of *FgLaeA* deletion and overexpression on the production of mycotoxins [trichothecenes (e.g. deoxynivalenol, DON; 15-acetyl DON, 15ADON) and zearalenone (ZEA)] in *F. graminearum* was determined using the luminescence reporter system, chemical analysis, and qRT-PCR. In all tests, the WT strain produced both DON and ZEA with concomitant gene expression levels under appropriate conditions, whereas toxin production in the Δ*FgLaeA* strains significantly decreased. Luminescence signal intensity from cell lysates extracted from FLTRI6Δ*FgLaeA* strains grown in AG medium for 6 days were at least 10-fold lower than those from the FLTRI6 strain, demonstrating that the *Tri6* transcript level significantly decreased due to Δ*FgLaeA*; no significant difference in luminescence was observed between FLTRI6 strains grown under dark and light conditions (Fig. 4A). Similarly, luminescence intensities for *ZEB2* expression in the FLZEB2Δ*FgLaeA* strain decreased ∼2-fold compared to FLZEB2 when grown in AG medium (Fig. 4B) and ∼6-fold when grown on a rice substrate for 2 weeks (data not shown). GC-MS analysis using extracts from the fungal rice culture confirmed that 15ADON and ZEA were detected in the Z3643 and add-back strains, whereas no detectable amount of 15ADON or a ∼30-fold-reduced level of ZEA was detected in the Δ*FgLaeA* strain derived from Z3643 (Fig. 5, Table S1). However, a *FgLaeA-*overexpression strain (OE8) produced ∼11-fold-increased amounts of 15ADON along with additional metabolites, such as butenolide and culmorin, which were not detected in Z3643; no ZEA levels were altered (Fig. 5, Table S1). The same effect of Δ*FgLaeA* on the DON and ZEA production was also confirmed using the other wild-type strain (9F1) of *F. graminearum* that produces a significant amount of culmorin [38] (Fig S2). In addition, qRT-PCR confirmed that the gene transcript levels for theses metabolites, *Tri6* (FGSG_03536) required for 15ADON and *CLM1* (FGSG_10397) for culmorin were drastically reduced in the Δ*FgLaeA* strain, but increased in the OE strain when grown in AG medium (Fig. 4C).

**Figure 6:**
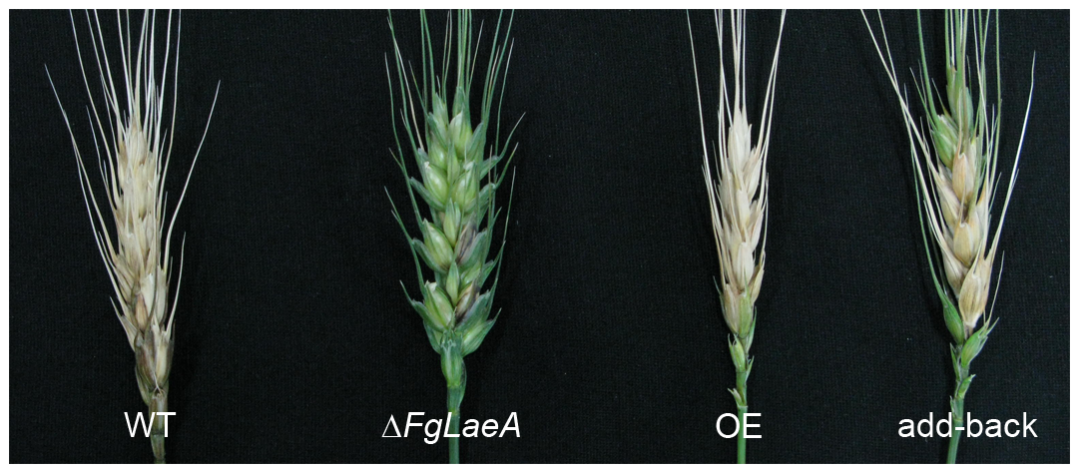
Virulence of the *F. graminearum* strains on wheat heads. The photographs were taken 14 days after inoculation. WT, wild-type strain Z3643; Δ*FgLaeA*, the Δ*FgLaeA* strain derived from Z3643; OE, the *FgLaeA*-overexpression strain derived from Z3643; add-back, the *FgLaeA-*add-back strain derived from the Δ*FgLaeA* strain.

In plant inoculation tests, the WT, OE, and add-back strains examined caused typical head blight leading to complete bleaching on wheat. The Δ*FgLaeA* strains were able to colonized inoculated spikelets, but rarely spread to the adjacent ones (Fig. 6).

### Expression Pattern of *FgLaeA*, *FgVeA* and *FgVelB* in the Fungal Strains

**Figure 7:**
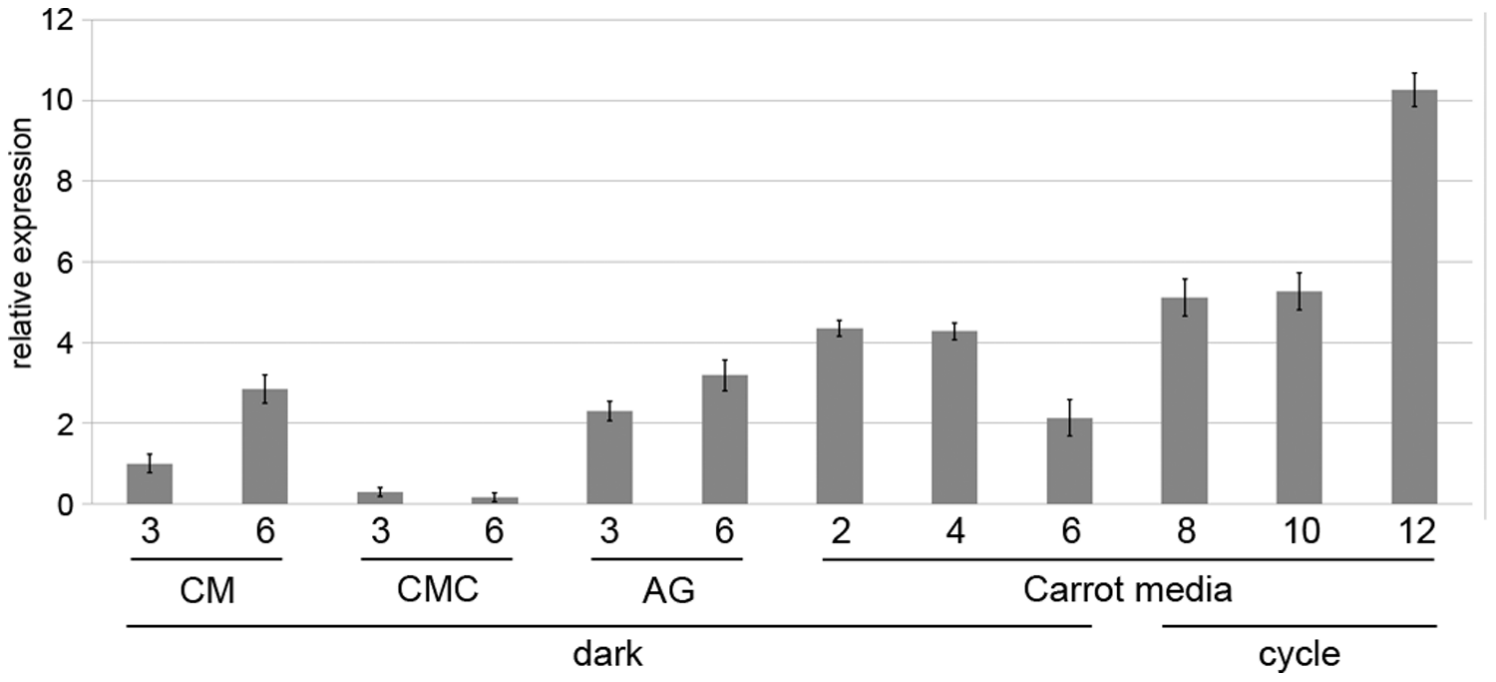
Expression of *FgLaeA* under various culture conditions. Relative *FgLaeA* transcript levels in the WT Z3643 strain grown in complete liquid medium for vegetative growth, CMC liquid medium for conidiation, AG liquid medium (AG) for trichothecene production, and on carrot agar for sexual development under dark or dark/light cycle conditions (cycle). Days of incubation following inoculation in each medium is shown in on the *x*-axis. *GzRPS16* (FGSG_09438.3) was used as an endogenous control for data normalization [51]. The amount of *FgLaeA* transcript from a 3-day-old sample in complete liquid medium was used as a reference.

qRT-PCR analysis revealed that the *FgLaeA* transcript was constitutively but weakly expressed in WT strain Z3643 during vegetative growth, perithecial formation on carrot agar, and trichothecene production in AG medium (Fig. 7). Except during conidiation in CMC medium, and 6 days after perithecia induction on carrot agar, *FgLaeA* transcript levels remained stable across all time points examined, staying within 1.5-fold relative to a reference gene (either *EF1A* or FGSG_09438) (Fig. 7). The constitutive low expression of *FgLaeA* under these conditions was clearly confirmed by RNA-seq analysis (unpublished data). The RPKM (reads per kilobase per million mapped reads) values of the *FgLaeA* transcripts accumulated under conditions for sexual development, trichothecene production, and vegetative hyphal growth were 4.3, 5.5, and 7.6, respectively. However, two other genes, *FgVeA* (FGSG_11955.3) and *FgVelB* (FGSG_01362.3), which may represent a FgVeA complex, showed sexual developmental-specific expression patterns and higher expression levels under all three conditions compared to *FgLaeA*. RPKMs for *FgVeA* and *FgVelB* were 325.9, 100.9, 126.4, and 630.9, 56.8, 33.0, respectively. Note that the *FgVelB* transcript, which significantly accumulated only on carrot agar for sexual development, could be clearly detected in a Northern blot hybridization [34]. In contrast, the *FgLaeA* transcript levels in two independent OE strains (OE7 and OE8) dramatically increased (up to ∼1,200-fold) compared to those in Z3643 during the entire growth conditions examined; those in the add-back strains were not significantly different from those in Z3643 (Fig. S3A).

Expressions of two genes (*FgVeA* and *FgVelB*) for putative members of the FgVeA complex were dramatically elevated in the Δ*FgLaeA* strain on carrot agar (under the sexual developmental stage); only the *FgVeA* transcript level was increased in CM medium (Fig. S3B).

### Expression of Mating-type (*MAT*) Genes in the Δ*FgLaeA* Strain under Sexual Development

To investigate a possible genetic cause for the early sexual induction in the Δ*FgLaeA* strain, we compared gene expression profiles at the mating-type (*MAT*) locus, the master regulator for sexual development, among the WT, Δ*FgLaeA,* OE, and add-back strains. qRT-PCR analysis confirmed that the transcript levels of two *MAT* genes (*MAT1-1-1* and *MAT1-2-1*, both of which are required for early sexual stage development in *F. graminearum* [39]), were elevated ∼2–3-fold in the Δ*FgLaeA* strain compared to the WT strain 24 h after perithecial induction on carrot agar (Fig. 3B); no changes were observed in the OE and add-back strains during the same time points for perithecial induction compared to those in WT (Fig. 3B). However, the other three *MAT* genes (*MAT1-1-2*, *MAT1-1-3*, and *MAT1-2-3*) showed no dramatic differences in transcript levels among the strains examined (data not shown).

### Differentially Expressed Genes (DEGs) in the Δ*FgLaeA* Strain under Trichothecene Production Conditions

To identify genes regulated under control of *FgLaeA* during trichothecene production, we performed RNA-seq analysis using total RNA extracted from mycelia of Δ*FgLaeA* and Z3643 strains grown in AG liquid medium for 60 h, an early induction stage for trichothecene production. Analysis of transcriptional profiles revealed a total of 799 genes differentially regulated ≥2-fold in the FLTRI6Δ*FgLaeA* strain compared to its WT progenitor FLTRI6; 444 and 355 genes were significantly down- and upregulated, respectively (Tables S2, S3, S4). Gene ontology (GO) analysis using blast2go (http://www.blast2go.com) revealed enrichment of DEGs associated with catalytic activity among GO molecular functions, and enrichment of DEGs associated with metabolic and cellular processes among GO biological processes (Fig. S4).

Searching against the conserved domain database (CDD) revealed that 332 of the DEGs (63.4%) found within the database contained enzymatic domains or were related to enzyme functions, including 72 genes (13.7%) involved in metabolism. In contrast, only 16 genes (3.0%) were identified as those probably involved in development and signal transduction (Table S5). In addition, 8 and 17 genes for transcription factors (TFs) were identified as down- and upregulated, respectively (Tables S2, S3, S4); however, none of the TFs differentially regulated in the FLTRI6Δ*FgLaeA* strain were associated with distinct functions based on gene deletion assays [40]. Unlike in the *A. nidulans* Δ*laeA* strain [21], three major members of a putative FgVeA complex, *FgVeA*, *FgVelB*, and *FgVosA* (FGSG_06774.3), were not significantly differentially regulated in the FLTRI6Δ*FgLaeA* strain in AG medium; *FgVeA* and *FgVosA* showed only 1.48- and 1.43-fold increases, respectively, compared to FLTRI6 (note that the transcript levels of both *FgVeA* and *FgVelB* were dramatically increased in the Δ*FgLaeA* strain under sexual development condition, confirmed by qRT-PCR).

To identify genes for secondary metabolites, we compared the DEGs against members of the 77 tentative functional gene clusters (TFCs) in *F. graminearum*, previously identified using five types of functional descriptions [41]. Gene member(s) belonging to 37 TFCs and 19 TFCs were identified in downregulated and upregulated genes in the FLTRI6Δ*FgLaeA* strain, respectively (Tables S2, S3, S4). Moreover, members of nine TFCs were found in both down- and upregulated genes without overlapping.

Among the downregulated genes, seven genes (*Tri3–Tri14*) in the *Tri* gene cluster for trichothecene biosynthesis were identified; *Tri101* (FGSG_07896.3), located outside the gene cluster, was not differentially expressed. Three polyketide synthase (*PKS*) genes including *PKS10,* responsible for biosynthesis of fusarin C, and two *PKS* genes (*PKS2* and *PKS14*) whose chemical products have not yet been identified, a non-ribosomal peptide synthetase gene (*NPS7*), and *CLM1* (FGSG_10397) for culmorin biosynthesis were also identified. However, all of these genes except *PKS10* showed very low RPKM values (<0.5) in the WT FLTRI6 strain; eight members of a putative *PKS10* cluster (FGSG_07798–07807) showed higher RPKM values ranging from 5.4 to 90.0 in FLTRI6. In addition to these key enzyme genes, three additional genes in the *PKS* cluster (*PKS5*, *PKS11*, and *PKS12* for aurofusarin), one *NPS* gene (*NPS12*), and one butenolide cluster gene were downregulated. Six genes with contiguous gene numbers (FGSG_10608–10614), which were not deduced as a TFC in the previous study [41], were also downregulated (Tables S2, S3, S4). In contrast, no key enzyme genes in any TFCs were identified among the upregulated genes, with the exception of *PKS7* (FGSG_08795.3).

Mapping of the DEGs onto the *F. graminearum* genome revealed the DEGs to be dispersed throughout the four chromosomes; however, they were not evenly distributed on each chromosome. The genomic positions of some DEGs seemed to be enriched in specific regions such as subtelomeres, or genome-wide locations of histone H3-lysine methylations (H3K7me3 or H3Kme2) (Freitag, personal communication) (Fig. S5).

### Comparison of DEGs in the Δ*FgLaeA* and Δ*FgVelB* Strains

**Figure 8:**
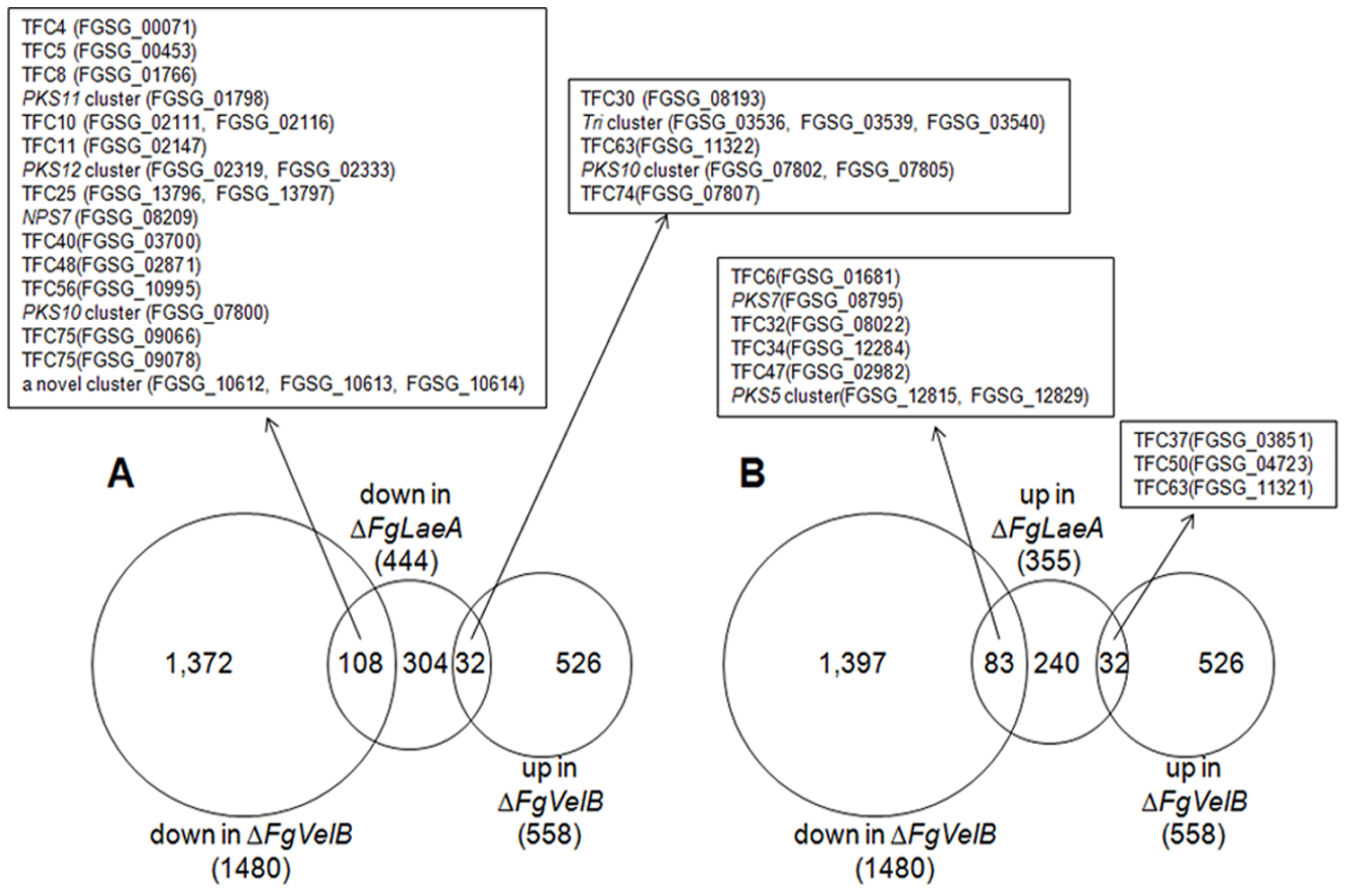
Number of genes expressed differentially in the *F. graminearum* Δ*FgLaeA* and Δ*FgVelB* strains. (A) Downregulated genes; (B) upregulated genes. The numbers of DEGs for each category is indicated in parentheses below the fungal strains. TFC, tentative functional cluster according to Lee (2010) [40].

We compared DEGs in the Δ*FgLaeA* strain under toxin production conditions with those obtained from the Δ*FgVelB* strain grown under sexual development conditions [34]. Although growth conditions for the gene deletion strains were different from each other, more than 30% of DEGs in the Δ*FgLaeA* strain overlapped with those from the Δ*FgVelB* strain (Fig. 8). Among 444 genes downregulated in the Δ*FgLaeA* strain, 108 (24.3%) and 32 (7.2%) genes were similarly downregulated and upregulated, respectively, in the Δ*FgVelB* strain; genes belonging to 16 TFCs were downregulated in both strains. Gene members of five TFCs including the *Tri* gene cluster were differentially expressed between strains, exhibiting downregulation in the Δ*FgLaeA* strain and upregulation in the Δ*FgVelB* strain. Similarly, 83 (23.3%) and 32 (9.0%) genes from the sets of down- and upregulated genes in the Δ*FgVelB* strain overlapped with those upregulated in the Δ*FgLaeA* strain, respectively (Fig. 8). Gene members of three TFCs were upregulated in both strains, while six other TFCs also showed differential expression between strains (Fig. 8).

### Cellular Localization of FgLaeA

To determine the cellular localization of the protein product of *FgLaeA* under various developmental stages, we constructed two additional transgenic fungal strains derived from the Δ*FgLaeA* strain, as well as the OE::*GFP-FgLaeA* strain described above. The Δ*FgLaeA*::*nGFP*-*FgLaeA* strain was generated by introducing an intact copy of *FgLaeA* fused to *GFP* under its native promoter into the Δ*FgLaeA* strain. The OE::*RFP-His::GFP-FgLaeA* strain was constructed by introducing the P*crp-GFP-FgLaeA* construct into the genome of a *F. graminearum* strain (H4::RFP) carrying a red fluorescence protein (RFP) fused to a histone gene [42]. The red pigmentation and perithecia formation pattern in both Δ*FgLaeA*::*nGFP*-*FgLaeA* and H4::RFP-OE::*GFP-FgLaeA* strains were restored to WT levels (Fig. 2). However, GFP expression in the former strains was not visualized by fluorescence microscopy.

**Figure 9:**
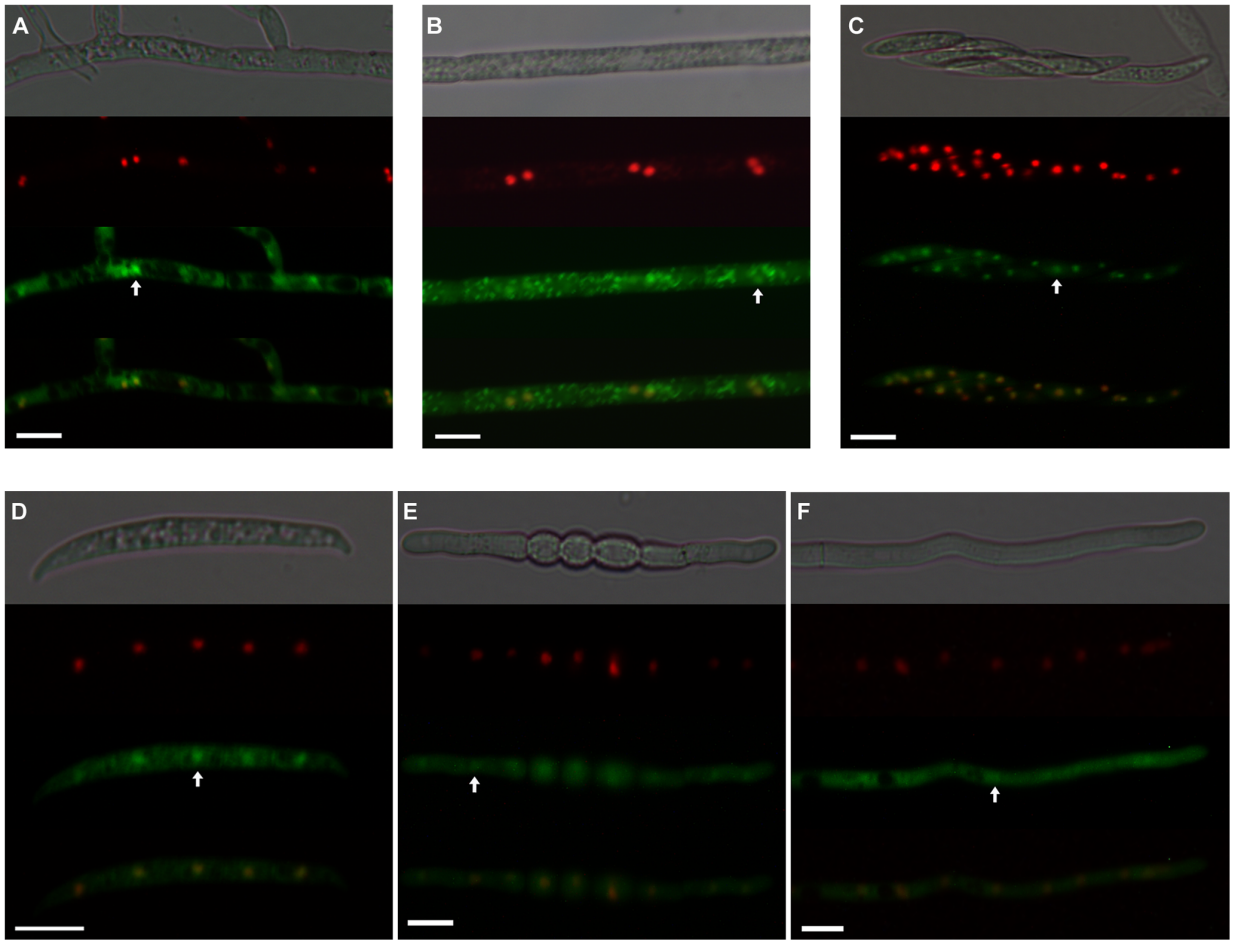
Expression of GFP in the *F. graminearum* OE::*GFP-FgLaeA* strain grown on CM plates. (A) Vegetative hyphae grown under dark/light cycle conditions; (B) those grown under constant dark conditions; (C) ascospore in ascus; (D) conidia; (E) conidium 3 h after germination; (F) apical tip of the germ tube after 7 h germination. Each picture consists of DIC, H4::RFP, GFP::FgLaeA and a merged picture of the RFP and GFP images, respectively. White arrows indicate the nuclei in each image. Size bar is 10 µm.

In both OE::*GFP-FgLaeA* and H4::RFP-OE::*GFP-FgLaeA* strains, GFP-FgLaeA commonly localized to both the nucleus and cytoplasm in fungal cells grown under all of stages examined, including newly formed conidia, germinating conidia, mature hyphae, and ascospores under both dark and light conditions (Fig. 9). Occasionally, nuclear enrichment of GFP signals was not obvious; instead, GFP expression was clearly seen in cellular compartments other than the nucleus (Fig. 9B).

### Protein Interactions between FgLaeA and FgVeA, FgLaeA and FgVelB, and FgVeA and FgVelB

To determine if the FgLaeA protein is a member of a possible FgVeA complex (i.e., a direct interaction occurred between FgLaeA and FgVeA), we employed the split luciferase complementation assay, a sensitive and efficient method of monitoring *in vivo* protein–protein interactions in filamentous ascomycetes [43]. For this assay, we generated plasmid vectors carrying the entire coding region of *FgLaeA* fused to a DNA region encoding an N-terminal fragment of luciferase (FgLaeA–nLuc) and the *FgVeA* fused to a C-terminal fragment (cLuc–FgVeA), respectively, and introduced either or both of them to the genome of Z3643. All cell lysates from negative controls grown in complete liquid medium, which were transgenic *F. graminearum* strains carrying a single empty vector expressing only nLuc or cLuc (pFNLucG or pFCLucH), a single-fused gene (*FNLuc–FgLaeA* or *FCLuc–FgVeA*), or the single fused gene along with the corresponding singly empty vector, exhibited only background levels of luminescence similar to WT strains (Table 1). In contrast, all fungal transformants co-expressing both of the fused proteins, FNLuc–FgLaeA and FCLuc–FgVeA, exhibited ∼1,000–2,300-fold increased luminescence relative to negative controls (Table 1). Similarly, we examined whether other *F. graminearum* LaeA-like proteins, including FgVIP1 described above, had the capacity to interact with FgVeA. No significant increase in luminescence intensity was found in the fungal strains co-expressing the FNLuc–FgVIP1 and FCLuc–FgVeA compared to the negative controls (Table 1). In addition, we examined the interactions between FgLaeA and FgVelB as well as between FgVeA and FgVelB. Transgenic *F. graminearum* strains co-expressing the *FNLuc-FgVelB* and *FCluc-FgVeA* constructs showed higher levels of luminescence compared to WT strains (Table 1), demonstrating the FgVeA-FgVelB interaction. Interestingly, the luminescence levels in the cell lysates from the fungal strains carrying both *FNLuc-VelB* and *FCLuc-FgLaeA* were also higher than those from the WT strains and negative controls although the luminescence levels for the FgLaeA-FgVelB interaction were much lower than those from the other fungal strains showing positive signals (Table 1).

**Table 1:** Luminescence activity^1^ in fungal cell lysates.

Strain	Interaction	Plasmids inserted	RLU^2^
Z3643	no	None	15-±6
cLUCnLUC	no	pFNLucG & pFCLucH	23±13
GzFNCS-1	FBP1-SKP1	pFNLuc-FBP1G &pFCLuc-SKP1H	19,815±7,268
VeALaeA-5	FgLaeA-FgVeA	pFNLuc-FgLaeAG &pFCLuc-FgVeAH	67,555±15,605
VeALaeA-8	FgLaeA-FgVeA	pFNLuc-FgLaeAG &pFCLuc-FgVeAH	28,849±5,341
VeAVelB-4	FgVeA-FgVelB	pFNLuc-FgVelBG &pFCLuc-FgVeAH	2,596±1,790
VeAVelB-5	FgVeA-FgVelB	pFNLuc-FgVelBG &pFCLuc-FgVeAH	2,786±1,543
VelBLaeA-1	FgLaeA-FgVelB	pFNLuc-FgVelBG &pFCLuc-FgLaeAH	249±94
VelBLaeA-4	FgLaeA-FgVelB	pFNLuc-FgVelBG &pFCLuc-FgLaeAH	505±294
VeAVIP1-6	FgVIP1-FgVeA	pFNLuc-FgVIP1G &pFCLuc-FgVeAH	29±6
VeAVIP1-9	FgVIP1-FgVeA	pFNLuc-FgVIP1G &pFCLuc-FgVeAH	39±4
LaeAN-1	FgLaeA-FN^3^	pFNLuc-FgLaeAG & pFCLucH	48±2
LaeAN-3	FgLaeA-FN	pFNLuc-FgLaeAG & pFCLucH	35±4
LaeAC-1	FC^3^-FgLaeA	pFNLucG & pFCLuc-FgLaeAH	28±3
VeAC-2	FC-FgVeA	pFNLucG & pFCLuc-FgVeAH	22±4
VeAC-3	FC-FgVeA	pFNLucG & pFCLuc-FgVeAH	28±8
VelBC-3	FC-FgVelB	pFNLucG & pFCLuc-FgVelBH	30±5
VIP1C-2	FgVIP1-FC	pFNLuc-FgVIP1G & pFCLucH	11±7
VIP1C-3	FgVIP1-FC	pFNLuc-FgVIP1G & pFCLucH	15±2

1 obtained from three replicates.

2 relative light units per microgram of protein per min.

3 FC: a C-terminal fragment of the firefly luciferase, FN: a N-terminal fragment.

### Development of a Firefly Luciferase Reporter System for Mycotoxin Production

To circumvent several problems in the chemical analysis of mycotoxin production, we developed a new reporter system for mycotoxin production in *F. graminearum* using firefly luciferase. A DNA construct carrying the firefly *Photinus pyralis* luciferase gene (designated *Fluc*) under control of a putative promoter region (∼1,000 bp) from either *TRI6* or *ZEB2* (transcription factors controlling the production of trichothecenes and zearalenone, respectively) was inserted into its native genomic region in *F. graminearum* strain Z3643 via homologous recombination (Fig. S6, S7). The single crossover integration events, confirmed by Southern hybridizations, resulted in fungal strains designated FLTRI6 and FLZEB2, respectively (Figs. S6, 7). No differences were seen between strains FLTRI6 and FLZEB2 and their WT progenitor (Z3643) for hyphal growth, pigmentation, conidiation, sexual development, and toxin production (data not shown). Strain FLTRI6 was grown in AG liquid medium, which favors trichothecene production [15], for 7 days. Luminescence signal intensities from cell lysates peaked at day 6, whereas only basal levels were detected in complete medium (CM; Fig S6C). Gas chromatography-mass spectrometry (GC-MS) analysis revealed that the FLTRI6 strain began producing both DON and 15ADON at day 4, and showed maximum levels of toxin production at day 7; no detectable level of trichothecenes were produced in complete liquid medium during the entire incubation periods (data not shown). Similarly, the luminescence signals from the FLZEB2 strain grown in SG liquid medium, which favors zearalenone production [14], increased up to day 8, but showed only basal level when grown in CM (Fig. S7C). Higher luminescence signals and toxin accumulations were detected on 2-week-old rice cultures of the FLZEB2 strain, a more favorable condition for the production of both toxins (data not shown). A similar pattern in time-course increases between luminescence signals and toxin accumulations from these strains demonstrated that both the FLTRI6 and FLZEB2 strains could be used as luminescence reporter systems for qualitative and quantitative determination of mycotoxin production in *F. graminearum*.

## Discussion

The results presented here clearly demonstrate that a *LaeA*-like gene (*FgLaeA*) of *F. graminearum* is involved in controlling developmental processes, secondary metabolism, and virulence on host plant. Reduced pigmentation of aerial mycelia in the Δ*FgLaeA* strains is one of the most common growth-related phenotype in the *LaeA*-deletion strains of the previously characterized filamentous fungi except *C. heterostrophus* [27], in which pigmentation was increased, even though the biosynthetic pathways for each polyketide pigment were different (Table 2). This phenotypic change could have been attributable to the role of *FgLaeA* in controlling secondary metabolism described below. However, the leaky pigmentation phenotype on agar plates indicates that *PKS12-*mediated aurofusarin production is not tightly regulated by *FgLaeA*, especially when hyphal elongation was physically blocked (e.g., by Petri dish wall or neighbor colonies on agar plates). The relatively minor effect of Δ*FgLaeA* on light-dependent asexual development (conidiation) under dark condition was also similar to those of *LaeA* homologs in other fungi, in which the conidiation mostly decreased (Table 2). However, no disturbance of light-dependent conidiation by either the absence or overproduction of *FgLaeA* implies that *FgLaeA* is not involved in a light-sensing pathway for conidiation in *F. graminearum*.

**Table 2:** Comparison of phenotypic changes^a^ caused by the *LaeA*-like gene deletion or overexpression in filamentous fungi.

Species	Hyphal growth & morphology	Pigmentation	Conidiation	Sexual development	Secondary metabolism^b^	Virulence	References
*A. nidulans*	similar to WT	loss	reduced	increased smaller, lowerfertile cleisothecia	reduced for ST and PN; **increased for** **PN, LOV, & monocolin J**	NA^c^	[20], [22], [25]
*A. fumigatus*	similar to WT; more hyphal mass	loss	reduced	ND^d^	reduced for gliotoxin	impaired	[20], [25]
*A. flavus*	similar to WT	loss	reduced; **increased on** **peanut**	no sclerotia; **increased**	reduced for AF, cyclopiazonic acid, &kojic acid; **increased for AF &** **additional metabolites**	NA^c^	[24]
*P. chrysogenum*	curly & hyperbranching hyphae	ND^d^	reduced, light-dependent	ND^d^	reduced for PN	NA^c^	[43]
*C. heterostrophus*	reduced growth; reducedtolerance to H_2_O_2_	increased; **reduced**	increased under vegetative	female-sterile;**female-sterile**	reduced for T-toxin in the dark;**increased for T-toxin in both**	reduced	[27]
*T. reesei*	similar to WT	loss	reduced; **increased;** **light-dependent**	ND^d^	loss of cellulases; **increased for** **cellulases**	NA^c^	[31], [32]
*F. fujikuroi*	similar to WT	slightly reduced;**increased**	reduced	ND^d^	reduced for GA; **increased for** **bikaverin**	reduced	[28]
*F. verticillioides*	ND^d^	ND^d^	ND^d^	ND^d^	reduced for bikaverin, fusaric acid,& fusarin C	ND^d^	[29]
*F. oxysporum*	increased growth rate; no aerialmycelium; reducedhydrophobicity	ND^d^	reduced	ND^d^	reduced for siderophores,beauvericin, & fusaric acidin the dark	reduced	[30]
*F. graminearum*	fewer aerial mycelium; reducedhydrophobicity; **similar to WT**	reduced or loss;**similar to WT**	reduced only in the dark;light-dependent;**similar to WT**	slightly increased;light-dependent**similar to WT**	reduced for DON, ZEA, CM, &fusarin C; light-independent;**increased for DON,** **BT, & CM**	reduced	this study

a phenotypes caused by *FgLaeA-*overexpression were shown in bold in the same column for each description.

b abbreviations for metabolites: PN, penicillin; LOV, lovastatin; AF, aflatoxins; GA, gibberellic acids; DON, deoxynivalenol; ZEA, zearalenone; BT, butenolide; CM, culmorin.

c not applicable;

d not determined.

In contrast to these conserved functions, an earlier induction of perithecia formation in the Δ*FgLaeA* strain (and recovery to WT levels in both add-back and OE strains) provided genetic evidence demonstrating a distinct role of *FgLaeA* during sexual development in *F. graminearum*. This phenotype could be comparable to the elevated, constitutive formation of fruiting bodies (cleistothecia) in the *A. nidulans* Δ*laeA* strain (Table 2), implying a conserved role of *LaeA* as a negative regulator for initiating sexual development in both fungi. However, the effect of gene deletions on completion of sexual development were different between the two species. The *A. nidulans* Δ*laeA* strain was not able to form Hülle cells, which nurse the young cleistothecia, leading to the production of smaller, less fertile cleistothecia [22]; no obvious impairments in maturation of fertile perithecia were observed in *F. graminearum.*

The other striking difference between *F. graminearum LaeA* and *LaeA* from other species is the effect of light on sexual development. In *A. nidulans*, cleistothecia formation occurs preferentially in the dark and is inhibited by light. This responsiveness, required for proper sexual development, is disrupted in Δ*laeA* strains, as elevated cleistothecia formation is observed even in the presence of light [22]. In contrast, the light effect on inducing sexual development in *F. graminearum* is completely opposite to that of *A. nidulans*; perithecia formation occurs exclusively under either light or dark/light cycle conditions, and is completely inhibited under dark condition. Even though the absence of *FgLaeA* enhanced sexual development, it did not affect light-dependent perithecia formation, suggesting that *FgLaeA* may play a role in repressing sexual developmental processes, especially during early stages. However, unlike *A. nidulans laeA*, *FgLaeA* is not involved in sensing light signals for initiating sexual development, which is similar to its role in regulating conidiation, as described above. The effect of *LaeA* deletion in *F. graminearum* was opposite that of *C. heterostrophus*, when Δ*Chlae1* caused female sterility, suggesting a role in positive regulation of sexual reproduction by *ChLAE* in this species [27]. Even though this phenotypic change in the Δ*FgLaeA* strain seems relatively subtle, the distinct role of *FgLaeA* in sexual development becomes more readily apparent when compared to phenotypic changes caused by the deletion of other velvet complex member genes in *F. graminearum* (e.g., Δ*FgVeA* and Δ*FgVelB*) [33]-[35]. Loss of *FgVeA* and *FgVelB* completely abolished the capability of *F. graminearum* to form perithecia, which is opposite to effect of Δ*FgLaeA*. Downregulation of three *MAT* genes in the Δ*FgVelB* strain, confirmed by a microarray analysis, is consistent with *FgVelB* as a positive regulator of *MAT* gene expression [34]. In contrast, increased *MAT* transcript levels in the Δ*FgLaeA* strain during early stage of perithecia formation, confirmed by qRT-PCR, could be attributable to the enhanced sexual development phenotype, indicating that *FgLaeA* controls *MAT* gene expression in a manner different from that of the other FgVeA complex members (i.e., independently of the FgVeA complex). In addition, upregulation of *FgVeA* and *FgVelB* in the Δ*FgLaeA* strain under perithecial induction stage, but not under toxin production stage also suggests a specific role for FgLaeA (as a negative regulator) in modulating FgVeA complex for sexual development, as proposed in *A. nidulans* [21], [22].

In contrast to developmental processes, secondary metabolism was significantly affected by Δ*FgLaeA* in *F. graminearum*, consistent with the role of *LaeA* homologs in many filamentous fungi (Table 2). Not only reduced production of several mycotoxins and their corresponding gene expression levels in the Δ*FgLaeA* strain, but increased production of these metabolites by *FgLaeA* overexpression clearly confirms *FgLaeA* as a positive regulator of secondary metabolite biosynthesis. In addition, RNA-seq analysis of a Δ*FgLaeA* strain (FLTRI6Δ*FgLaeA*) grown under conditions favoring trichothecene production supports the role of *FgLaeA* as a global regulator for secondary metabolite production, including these mycotoxins. Among the DEGs in the Δ*FgLaeA* strain were genes for key enzymes (e.g., PKS, NRPS, terpenoid synthase, transporters, and cytochrome P450) and other members *F. graminearum* TFCs for secondary metabolites [41]. These genes were identified at a high frequency (56 of 77), indicating that these TFCs may be globally regulated by *FgLaeA*, although in many TFCs, only a small number of genes were found to be differentially expressed. Further RNA-seq analyses using an *FgLaeA*-overexpressing strain, as well as the Δ*FgLaeA* strain under different conditions favoring other metabolites, would be necessary to confirm a global role for *FgLaeA* in controlling secondary metabolite production in *F. graminearum*. Localization of some DEGs in subtelomeric or H3K7 methylation regions of each of the *F. graminearum* chromosomes would support a role for *FgLaeA* in chromatin-based regulation of gene expression for secondary metabolites, as proposed in *Aspergillus* species [44]. Taken together, these data provide conclusive evidence that *FgLaeA* has a conserved role in secondary metabolism at the global level in *F. graminearum*, as demonstrated in other species of *Fusarium* (*F. fujikuroi*, *F. verticillioides*, and *F. oxysporum*), as well as distantly related species (Table 2).

Fewer symptoms on wheat displayed in the Δ*FgLaeA* strain could be attributed to impaired ability of this strain to produce a secondary metabolite required for disease development, such as trichothecenes [12], which is comparable to the effect of other fungal *LaeA* genes on the production of secondary metabolites required for virulence in *A. fumigatus* [25], *C. heterostrophus* [27], and *F. fujikuroi* [28]. However, more attenuated symptoms caused by Δ*FgLaeA* than those by the gene disruption for trichothecene biosynthesis [12] suggests that *FgLaeA* controls additional secondary metabolisms and/or regulatory pathways essential for disease development in *F. graminearum*.

The next question we wanted to ask was whether the regulatory roles of *FgLaeA* described above are associated with those of other members of the putative FgVeA complex, as in *A. nidulans* and other species [21], [23], [27], [28], [30], [45]. To address this question, we would need to demonstrate a direct interaction between FgLaeA and FgVeA in *F. graminearum*, similar to that seen in *A. nidulans*, *Penicillium chrysogenum*, and *F. fujikuroi* [21], [28], [45], in which protein–protein interactions were confirmed by either yeast two-hybrid or bimolecular fluorescence complementation (BiFC) analysis. A previous study showed no interaction between FgLaeA and FgVeA using a yeast two-hybrid method [33]. Evidence provided in this study directly contradicts this finding; we demonstrated a clear *in vivo* protein–protein interaction between FgLaeA and FgVeA in *F. graminearum* using a newly developed split-luciferase complementation assay [43], implying that FgLaeA plays a role as a member of the FgVeA complex. In addition, the interaction between FgVeA and FgVelB, which was also confirmed by split luciferase complementaion, clearly supports the existence of the heterotrimeric FgLaeA–FgVeA–FgVelB complex in *F. graminearum*. Interestingly, positive (but lower) luminescence signals from the interaction between FgLaeA and FgVelB, which has not been intensively investigated in other fungi except *A. nidulans* [21], [22], may suggest a possibility for the direct interaction between these two protein in *F. graminearum*. However, it is also possible that FgVeA acts as a bridge to bring the FNLuc-FgVelB and FCLuc-FgLaeA into close proximity, as shown in *A. nidulans* [22], which leads to a partial complementation of split luciferase domains. Furthermore, more than 30% of DEGs in the Δ*FgLaeA* strain overlapped with those in the Δ*FgVelB* strain despite the difference in culture conditions for each gene deletion strain (Fig. 8). These overlapping DEGs may be common targets of both FgLaeA and FgVelB, or the FgLaeA–FgVeA–FgVelB complex. The presence of DEGs displaying opposite gene expression patterns between two strains (i.e., 32 genes downregulated the Δ*FgLaeA* strain and upregulated in the Δ*FgVelB* strain, and 83 genes upregulated in the Δ*FglaeA* strain and downregulated the Δ*FgVelB* strain) suggests their expression may be differentially controlled by the FgVeA complex, which is growth condition-dependent. For example, the trichothecene gene cluster is positively controlled by the FgVeA complex in agmatine liquid medium, while negatively controlled during the sexual development, as previously suggested [34].

In addition to protein–protein interactions, constitutive nuclear localization of LaeA homologs would be important evidence regarding the regulatory role of LaeA. Nuclear localization of the OE::*GFP-FgLaeA* product during various developmental stages, such as mature hyphae, conidia, germinating conidia, and ascospores, regardless of light signals, was clearly demonstrated in this study. However, exclusive nuclear enrichment of a LaeA–GFP signal in *A. nidulans*, as evidenced based on the nuclear/cytoplasmic GFP signal ratio [21], was not evident in *F. graminearum* (Fig. 9), in which significant levels of GFP expression were visualized in the cytoplasm as well as in the nucleus. This localization could be inferred based on nonspecific accumulation of the excessively producing GFP–FgLaeA in the cytoplasm. However, considering that the *LaeA–GFP* constructs used in cellular localization analyses in *A. nidulans* [20], [21] and *F. fujikuroi* [28] were expressed under the control of strong promoters such as those for *alcA*, *niiA*, and *gpd* in *A. nidulans*, the cytoplasmic distribution of GFP–FgLaeA may be not an artifact, but reflect a distinct function of FgLaeA compared to other fungi.

In conclusion, *FgLaeA* plays important roles in controlling secondary metabolism, various developmental processes, and virulence in *F. graminearum*, not only through functions conserved across several filamentous fungi (e.g., as a member of the FgVeA complex), but also through distinct additional functions (e.g., FgVeA complex-independent), which may reflect the unique life style of *F. graminearum*. Looking beyond *FgLaeA*, the efficiency and usefulness of the firefly luciferase reporter system for mycotoxin production developed here was clearly demonstrated in this study. This system has a potential for application in other investigations, e.g., using a recipient strain for an insertional mutagenesis to identify a novel gene controlling the expression of *Tri6* or *ZEB2*.

## Materials and Methods

### Fungal Strains and Culture Conditions

The *F. graminearum* WT strains Z3643, provided by Dr. Robert L. Bowden (USDA-ARS Plant Science and Entomology Research Unit, Manhattan, KS, USA), and 9F1 [38] belongs to lineage 7 of the *F. graminearum* species complex [7], [46]. T43ΔM2-2, acting as a female in an outcross, is a *MAT1-2-1-*deleted strain derived from Z3643 [47]. A *F. graminearum* transgenic strain carrying histone H4 fused with RFP [42] was used for the nuclear localization of FgLaeA. The WT and transgenic strains derived from Z3643 were stored in 20% glycerol at –70°C. Conidiation was induced in CMC [48] liquid medium. For sexual development, strains were inoculated and incubated on carrot agar as previously described [3], [47]. For genomic DNA extraction, each strain was grown in 50 mL CM [3] at 25°C for 72 h on a rotary shaker (150 rpm). For trichothecene production and total RNA extraction, 1 mL conidial suspension (10^5^/mL) of each strain was inoculated into the AG-amended liquid medium [49], as previously described. For zearalenone production and total RNA extraction, fungal strains were grown in SG liquid medium or on rice substrate, as previously described [14].

### Nucleic Acid Manipulations and Primers

Fungal genomic DNA was prepared as described previously [3], [50], and total RNA was extracted from mycelia using an Easy-Spin Total RNA Extraction kit (Intron Biotech, Seongnam, Korea) according to the manufacturer’s instructions. Other general procedures for nucleic acid manipulations were performed as described previously [51]. DNA gel blots were hybridized with biotinylated DNA probes prepared using the BioPrime DNA labeling system (Invitrogen, Carlsbad, CA, USA) and developed using the BrightStar® BioDetect™ Kit (Ambion, Austin, TX, USA). All PCR primers used in this study (Table S5) were synthesized by the Bioneer Corporation (Chungwon, Korea). qRT-PCR was performed with SYBR Green Super Mix (Bio-Rad, Hercules, CA, USA) using first-strand cDNA synthesized from total RNA; amplification efficiencies of all genes were determined as previously described [52]. Gene expression was measured in three biological replicates from each time point. *GzRPS16* (FGSG_09438.3) and *EF1A* (FGSG_08811.3) were used as endogenous controls for data normalization [52].

### Mycotoxin Analysis, Virulence Test, and Luciferase Assay

*F. graminearum* strains were grown in 20 mL AG liquid medium for 6 days for trichothecene production and extracted as described previously [53]. For production of both trichothecenes and zearalenone, and other metabolites, fungal strains were grown in triplicate on 40 g of rice substrate inoculated with spores washed from V8 juice plate for 7 days in the dark at 25°C. The fungal cultures were extracted on a rotary shaker with 100 ml ethyl acetate, dried on a rotoevaporator, and re-dissolved in 20 ml acetonitrile-water (86∶14). Five milliliter of this solution was cleaned with a Romer MycoSep225® Trich column, dried under a stream of nitrogen, and re-dissolved in 1 ml methanol. GC-MS analysis was performed on a HP 6890 has chromatograph fitted with HP-5MS (30 m×0.25 mm film thickness) and a 5973 mass detector. The carrier gas was helium with a 20∶1 split ratio and a 20 ml/min slot flow. The column was held at 120°C at injection, heated to 260°C at 25°C/min and held at 260°C for 14 min. Compounds were identified by comparison of GC retention time and mass spectral fragmentation with those of standard compounds.

The virulence of fungal strains was determined on wheat heads. Briefly, conidia of each strain were harvested from CMC culture, suspended in sterile water (1×10^6^ conidia/ml), and injected into a floret in the basal spikelet of the wheat (cv. Eunpamil) head at mid-anthesis. The plants were placed in a greenhouse for two weeks after a 3-day incubation in a humidity chamber.

Luciferase activity in cell lysates from fungal strains was measured using GloMax® 96 Microplate Luminometer (Promega) as previously described [43].

### RNA-seq Analysis

PolyA tailed transcript RNA (mRNA) was enriched from total RNAs prescreened for quality using Oligo dT beads (TruSeq RNA Sample Prep. Kit, FC-122-1001,1002; Illumina, San Diego, CA, USA) and fragmented for cDNA synthesis by reverse transcriptase (SuperScript II Reverse Transcriptase, part no. 18064-014; Invitrogen). The synthesized cDNA underwent end-repair and subsequent 3′ A tailing for adaptor ligation (TruSeq RNA Sample Prep. Kit, FC-122-1001,1002; Illumina). After amplification of cDNA with adaptors, 300–400-bp cDNA fragments were finally selected. Paired-end sequencing of the amplified cDNA library was performed using the HiSeq2000 platform [TruSeq SBS Kit v3–HS (200 cycles), ILFC-401-3001; Illumina). RNA-seq reads were aligned to the *F. graminearum* genome sequences available from the Broad Institute (http://www.broadinstitute.org/annotation/genome/fusarium_group/MultiDownloads.html) and normalized to RPKM values for genes by using the CLC Genomics Workbench RNA-seq data analysis pipeline according to the developer’s instructions. RPKM values from each strain were used for identification of DEG sets. The RNA-seq data have been deposited in the NCBI Short Read Archive (http://www.ncbi.nlm.nih.gov/sra; SRP022085).

### Constructions of Vectors and Fusion PCR Products for Fungal Transformation

The DNA construct for deletion of *FgLaeA* from the genomes of *F. graminearum* strains Z3643, FLTRI6, and FLZEB2 was created using a split-marker recombination procedure as previously described [39], [54]. The 5′ and 3′ flanking regions of the *FgLaeA* ORF were amplified with the primer pairs 657for5/657revtail5 and 657fortail3/657rev3, respectively, fused to the geneticin resistance gene (*gen*) cassette, which was amplified from pII99 [55] with the primers Gen-for and Gen-rev, in the second round of PCR, and used as a template to generate split markers with the new nested primer sets, 657nest5/Gen-revN and 657nest3/Gen-forN, respectively (Table S6).

DNA plasmids carrying *FgLaeA* fused to *GFP* under control of the *cryparin* gene (*Crp*) promoter from *C. parasitica* [36] (P*Crp-GFP-FgLaeA*), and the same construct under control of the native promoter region of *FgLaeA* (Pnative-*GFP-FgLaeA*), were generated using a double-joint PCR [56]. For the P*Crp–GFP–FgLaeA* construct, three amplicons were amplified from pCHPH1 [36], pIGPAPA [47], and Z3643 with the primer pairs Pcrp_for/Pcrp-GFP tai/rev, GFP_for/GFP_rev, and LaeA-GFP tail/LaeA rev, respectively, mixed in a 1∶1:3 molar ratio, and finally fused together using the nested primer set Pcrp_for and LaeA 3rd fusion. For Pnative-*GFP-FgLaeA,* the 1.0-kb native promoter region of *FgLaeA* was amplified from Z3643 with primers pLaeA for and pLaeA rev. The final fusion product was amplified with the nested primer set pLaeA for and LaeA 3rd fusion. Each of these two fusion PCR products was cloned into pGEMT (Promega), creating pP*Crp–GFP–FgLaeA* and pPnative–*GFP–FgLaeA*, respectively. The DNA plasmid (pPnative–*FgLaeA*) used in generating the add-back strain was constructed by amplification of a 3.2-kb *FgLaeA* region including the *FgLaeA* ORF with its 5′ (0.9 kb) and 3′ (1.0 kb) flanking regions with primers LaeA5R and LaeA3F, followed by cloning into pGEMT.

For insertion of the firefly luciferase gene (*FLuc*) under control of the putative promoter region of *Tri6*, a 1.8-kb fragment upstream of the 5′ end of *Tri* ORF was fused to the *FLuc* ORF (1.6 kb) using a single-joint PCR method [56]. The promoter region and *FLuc* were amplified from the genomic DNA of Z3643 and the plasmid DNA pSPluc+NF fusion vector (Promega, Madison, WI, USA) with primer pairs Tri6SPL5/Tri6Luc3 and SPluc5/SPluc3 (Table S6), respectively. The two amplicons were mixed in a 1∶1 molar ratio and fused in a second round of PCR, followed by a third round of PCR using the nested primer set SPLuc5nest and TSPluc3nest (Table S6). The amplified product was cloned into pGEMT (Promega), creating pPTri6-FLuc. A *Sal*I-digested *hygB* cassette, derived from pBCATPH, was cloned into pPTri6-FLuc, resulting in a 8.9-kb plasmid pPTri6-FLuc-H. Similarly, the plasmid DNA carrying the *FLuc* gene fused to a promoter of *ZEB2* was constructed as described above. The *ZEB2* promoter region (1.8 kb) was amplified from Z3643 with the primers ZEBSPLuc5 and ZEBSPLuc3 for the first round of PCR and fused to the *FLuc* PCR product in the second round, followed by a third round with primers ZSLuc5nest and SPLuc3nest. Using the same cloning procedure described above, the final vector pPZEB2–Fluc-H was constructed.

The amplified fusion products or constructed DNA plasmids were added into the protoplasts of WT *F. graminearum* strains for transformation [39], [57]; pP*Crp–GFP–FgLaeA*, pPnative–*GFP–FgLaeA*, and pPnative–*FgLaeA* were added into the Δ*FgLaeA* strain along with pII99 carrying *gen*, respectively, for co-transformation.

### Split Luciferase Complementation Assay

For split luciferase complementation, the coding regions of the *FgLaeA* and *FgVIP1* genes, which were amplified from total RNA of Z3643 with the primer pairs LaeAnLuc-for2/LaeA-inrev3 and VIP1-forNT/VIP1-revNT, respectively, were introduced into the *Sal*I site of the DNA plasmid pFNLucG [43] carrying an N-terminal fragment of *FLuc* and *gen* using the In-Fusion® HD Cloning Kit (Clontech, Mountain View, CA, USA) as previously described [43], creating pFNLuc-FgLaeAG and pFNLuc-FgVIP1G (Table 1), respectively. Similarly, the cDNA of *FgVeA* (FGSG_11956.3) was cloned into pFCLucH [43] carrying a C-terminal fragment of *FLuc* and *hygB*, generating pFCLuc–FgVeAH (Table 1). The DNA plasmid pFCLuc–FgVeAH was added into the protoplasts of Z3643 along with either pFNLuc–FgLaeAG or pFNLuc–FgVIP1G for selection of fungal transformants carrying both plasmids. Luciferase activity was measured from the cell lysates of the transformants grown in CM liquid medium for 3 days, as previously described [43]. As a positive control, we included the transgenic *F. graminearum* GzFNCS-1 strain showing high luciferase activity driven by an *in vivo* protein–protein interaction between FBP1 and SKP1, fused to NLuc and CLuc, respectively [43]. The experiment was repeated three times.

### Microscopic Observations

Microscopic observations were performed using an image analysis system consisting of a microscope (Leica DM 2000) with attached digital camera (Leica DFC 550) and a computer.

## Supporting Information

Figure S1 (A) Pigmentation of the Δ*FgLaeA* strain in the marginal regions between different growing colonies grown on CM agar plate, and (B) when grown in CM liquid medium. Left and right, WT and Δ*FgLaeA* strain, respectively.
(TIF)

Figure S2 **Reconstructed ion chromatograms of rice culture extracts from the fungal strains derived from 9F1.** WT (9F1), the wild-type 9F1 strain; Δ*FgLaeA*, a 9F1 Δ*FgLaeA* strain derived from 9F1; add-back, a 9F1 *FgLaeA*-add-back strain derived from the 9F1Δ*FgLaeA* strain. A, 15ADON; C, culmorin; Z, zearalenone.
(TIF)

Figure S3 **Expression of** ***FgLaeA, FgVeA*** **and** ***FgVelB*** **in various** ***F. gramineaum*** **strains.** (A) Relative transcript levels of *FgLaeA* in the *FgLaeA-*overexpression and add-back strains, and (B) those of *FgVeA* and *FgVelB* in the Δ*FgLaeA* strain derived from Z3643, which were grown in CM liquid medium for vegetative growth, CMC liquid medium for conidiation, AG liquid medium for trichothecene production, and on carrot agar for sexual development. Days of incubation following inoculation in each medium are shown in on the *x*-axis. cycle, 12 h-dark/12 h-light cycle. The amounts of *FgVeA* and *FgVelB* transcripts from a 6-day-old sample in AG liquid medium were used as references in (A) and (B), respectively.
(TIF)

Figure S4 **Gene ontology analysis of DEGs in the Δ** ***FgLaeA*** **strain grown in AG liquid medium for 60 h.**
(TIF)

Figure S5 **Distribution of DEGs in the Δ** ***FgLaeA*** **strain on each chromosome of the** ***F. graminearum*** **PH-1 strain.** The genomic locations of histone 3 lysine methylations (H3K7me3 and H3K4me2) were indicated by thin black and red bars, respectively, below the positions of DEGs.
(TIF)

Figure S6 **Generation of a firefly luciferase reporter system for trichothecene production.** (A) Scheme for the insertion of the *FLuc* gene under control of a promoter region of *Tri6* into the genome of the *F. graminearum* Z3643 (WT) strain by homologous recombination. (B) *Sal*I-digested genomic DNA gel blot probed with the entire vector. Lane 1, WT strain; lane 2, the FLTRI6 strain. DNA size markers are indicated on the left side of the gel. (C) Luminescence signals in the cell lysate from FLTRI6 grown in AG and complete liquid media, respectively. Days postinoculation (DPI) are indicated below the days of incubation.
(TIF)

Figure S7 **Generation of firefly luciferase reporter system for zearalenone production.** (A) Scheme for the insertion of the *FLuc* gene under control of a *ZEB2* promoter into the genome of the *F. graminearum* Z3643 (WT) strain by homologous recombination. (B) *Sal*I-digested genomic DNA gel blot probed with the entire vector. Lane 1, WT strain; lanes 2 and 3, the FLZEB2 strains. DNA size markers are indicated on the left side of the gel. (C) Luminescence signals in the cell lysate from a FLZEB2 strain grown in SG and complete liquid media, respectively. Days postinoculation (DPI) are indicated below the days of incubation. Asterisks above the bars indicate a significant difference between two culture conditions for each DPI according to Tukey’s test.
(TIF)

Table S1 **Quantification of 15ADON and ZEA levels in fungal rice culture extracts.**
(XLSX)

Table S2 **Genes down-regulated in the Δ** ***FgLaeA*** **strain grown in agmatine-amended liquid medium for 60 h.**
(XLSX)

Table S3 **Genes up-regulated in the Δ** ***FgLaeA*** **strain grown in agmatine-amended liquid medium.**
(XLSX)

Table S4 **DEGs belonging to tentative functional gene clusters from the Δ** ***FgLaeA*** **strain.**
(XLSX)

Table S5 **Grouping of differentially expressed genes in the Δ** ***FgLaeA*** **strain by functional domains.**
(XLSX)

Table S6 **Primers used in this study.**
(DOCX)
