# Supplementary figures and images for: Functional Roles of FgLaeA in Controlling Secondary Metabolism, Sexual Development, and Virulence in Fusarium graminearum
Source: PLoS One. 2013 Jul 16;8(7):e68441. doi: 10.1371/journal.pone.0068441 (PMC3713025; doi:10.1371/journal.pone.0068441)

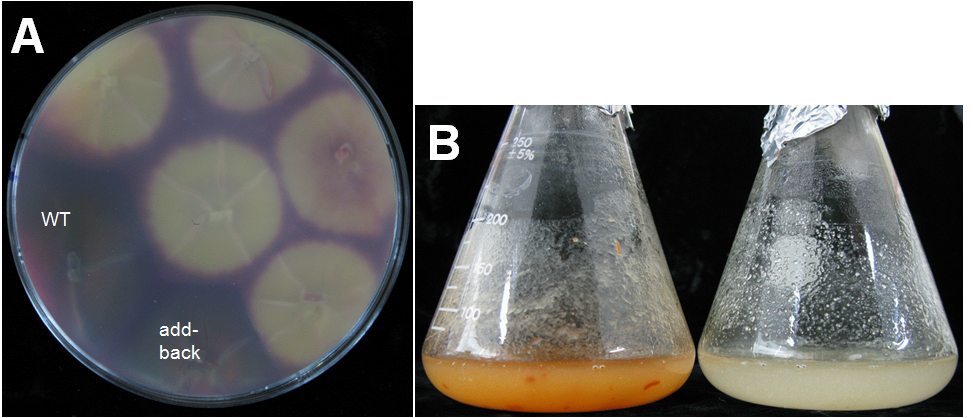

Supplement: Figure S1 — (A) Pigmentation of the ΔFgLaeA strain in the marginal regions between different growing colonies grown on CM agar plate, and (B) when grown in CM liquid medium. Left and right, WT and ΔFgLaeA strain, respectively. (TIF) [file pone.0068441.s001.tif]

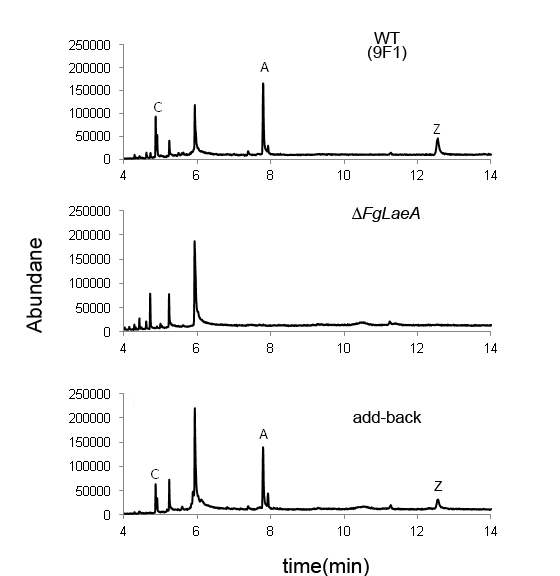

Supplement: Figure S2 — Reconstructed ion chromatograms of rice culture extracts from the fungal strains derived from 9F1. WT (9F1), the wild-type 9F1 strain; ΔFgLaeA, a 9F1 ΔFgLaeA strain derived from 9F1; add-back, a 9F1 FgLaeA-add-back strain derived from the 9F1ΔFgLaeA strain. A, 15ADON; C, culmorin; Z, zearalenone. (TIF) [file pone.0068441.s002.tif]

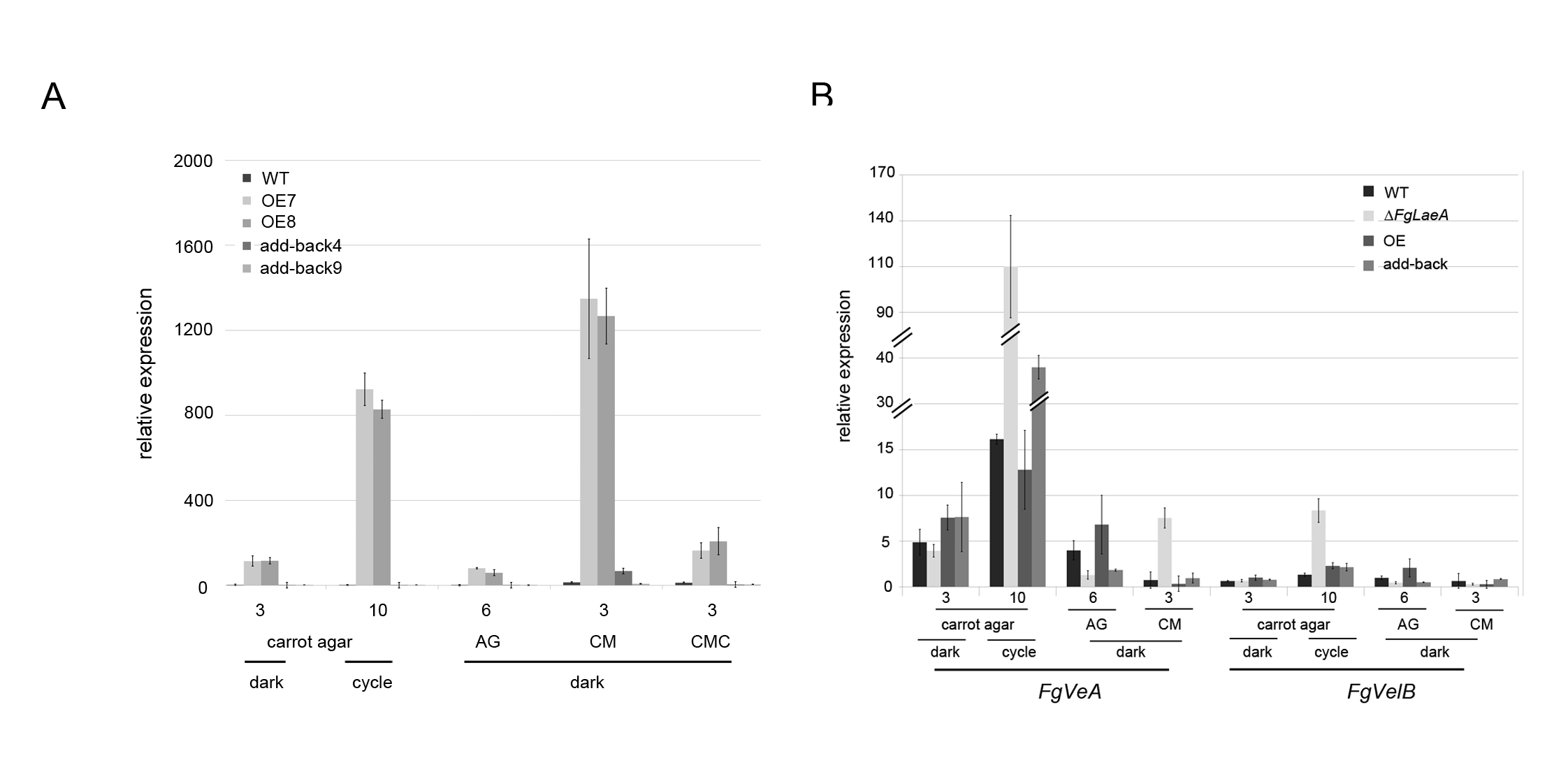

Supplement: Figure S3 — Expression of FgLaeA, FgVeA and FgVelB in various F. gramineaum strains. (A) Relative transcript levels of FgLaeA in the FgLaeA-overexpression and add-back strains, and (B) those of FgVeA and FgVelB in the ΔFgLaeA strain derived from Z3643, which were grown in CM liquid medium for vegetative growth, CMC liquid medium for conidiation, AG liquid medium for trichothecene production, and on carrot agar for sexual development. Days of incubation following inoculation in each medium are shown in on the x-axis. cycle, 12 h-dark/12 h-light cycle. The amounts of FgVeA and FgVelB transcripts from a 6-day-old sample in AG liquid medium were used as references in (A) and (B), respectively. (TIF) [file pone.0068441.s003.tif]

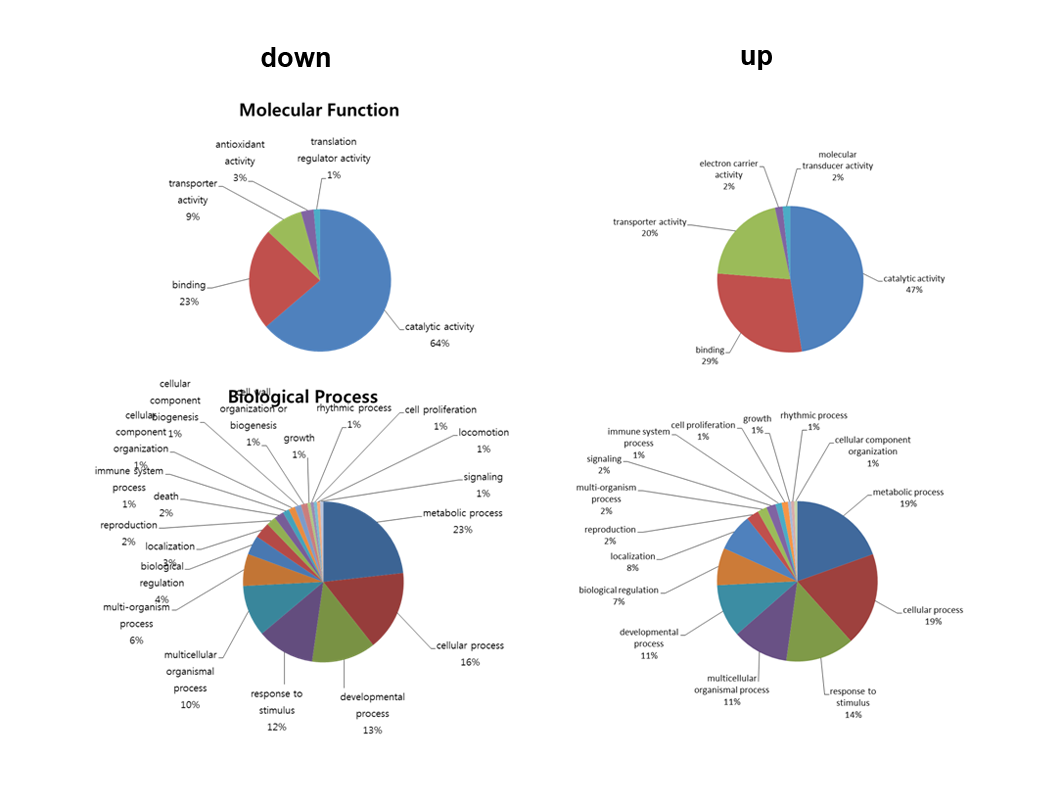

Supplement: Figure S4 — Gene ontology analysis of DEGs in the Δ FgLaeA strain grown in AG liquid medium for 60 h. (TIF) [file pone.0068441.s004.tif]

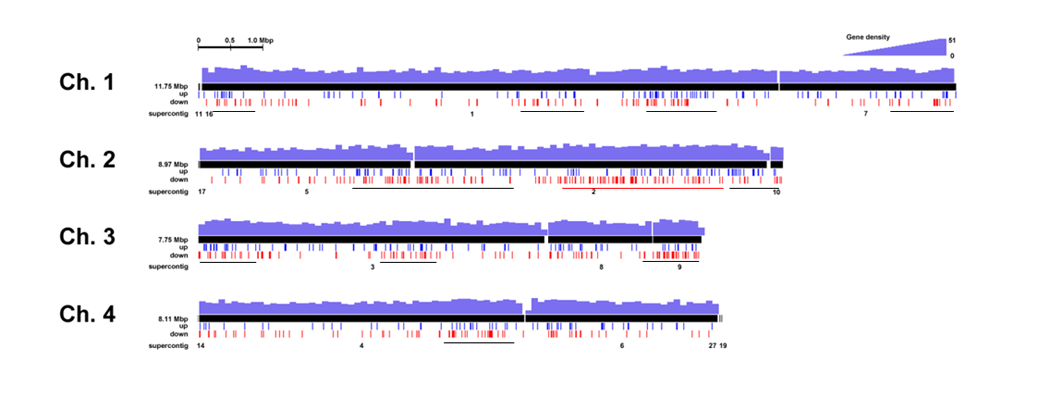

Supplement: Figure S5 — Distribution of DEGs in the Δ FgLaeA strain on each chromosome of the F. graminearum PH-1 strain. The genomic locations of histone 3 lysine methylations (H3K7me3 and H3K4me2) were indicated by thin black and red bars, respectively, below the positions of DEGs. (TIF) [file pone.0068441.s005.tif]

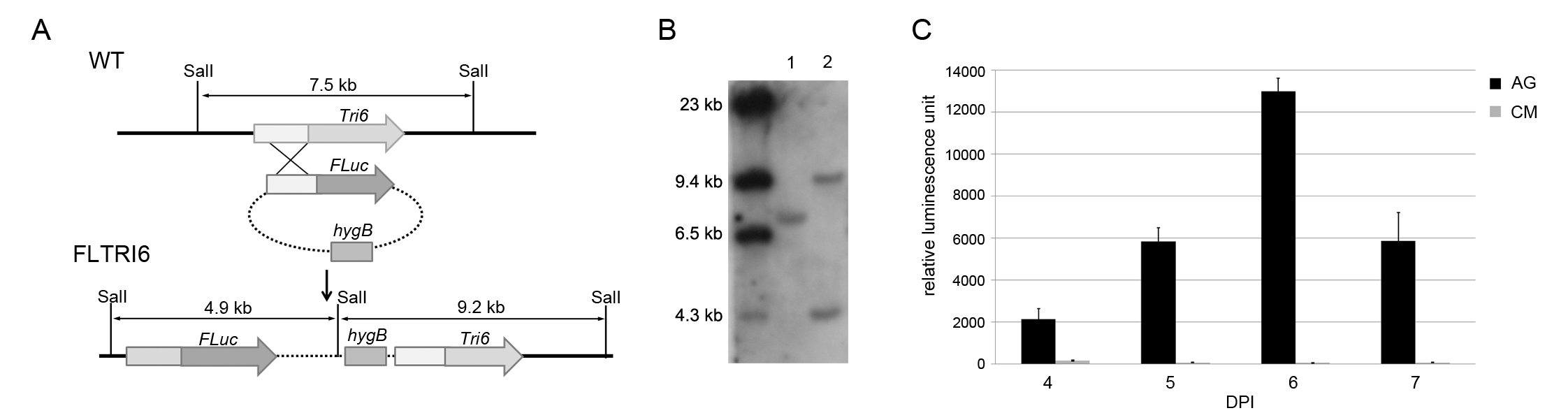

Supplement: Figure S6 — Generation of a firefly luciferase reporter system for trichothecene production. (A) Scheme for the insertion of the FLuc gene under control of a promoter region of Tri6 into the genome of the F. graminearum Z3643 (WT) strain by homologous recombination. (B) SalI-digested genomic DNA gel blot probed with the entire vector. Lane 1, WT strain; lane 2, the FLTRI6 strain. DNA size markers are indicated on the left side of the gel. (C) Luminescence signals in the cell lysate from FLTRI6 grown in AG and complete liquid media, respectively. Days postinoculation (DPI) are indicated below the days of incubation. (TIF) [file pone.0068441.s006.tif]

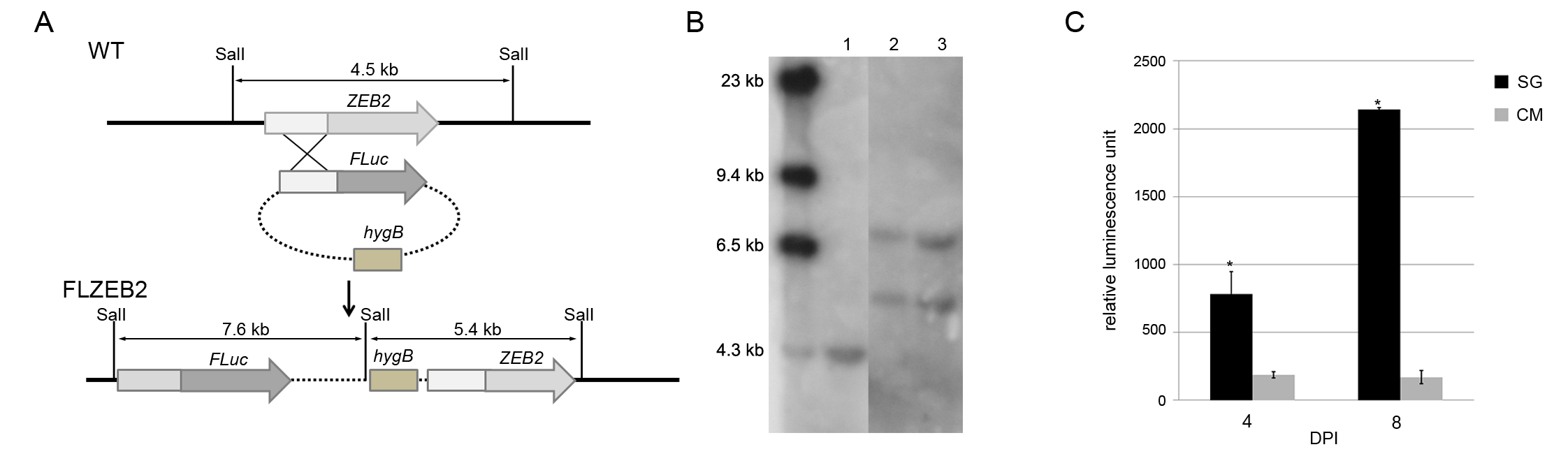

Supplement: Figure S7 — Generation of firefly luciferase reporter system for zearalenone production. (A) Scheme for the insertion of the FLuc gene under control of a ZEB2 promoter into the genome of the F. graminearum Z3643 (WT) strain by homologous recombination. (B) SalI-digested genomic DNA gel blot probed with the entire vector. Lane 1, WT strain; lanes 2 and 3, the FLZEB2 strains. DNA size markers are indicated on the left side of the gel. (C) Luminescence signals in the cell lysate from a FLZEB2 strain grown in SG and complete liquid media, respectively. Days postinoculation (DPI) are indicated below the days of incubation. Asterisks above the bars indicate a significant difference between two culture conditions for each DPI according to Tukey’s test. (TIF) [file pone.0068441.s007.tif]
