# Supplementary material for: Functional Roles of FgLaeA in Controlling Secondary Metabolism, Sexual Development, and Virulence in Fusarium graminearum
Source: PLoS One. 2013 Jul 16;8(7):e68441. doi: 10.1371/journal.pone.0068441 (PMC3713025; doi:10.1371/journal.pone.0068441)
Supplement: Table S6 — Primers used in this study. (DOCX) [file pone.0068441.s013.docx]

| **Table S6. Primers used in this study** | | | |
| --- | --- | --- | --- |
| Name | Sequence(5'→3') | Purpose | |
| Tri6SPLuc5 | TAGTCTCAGTACCAGCAAACAGGATAA | for FLTRI6 | |
| Tri6SPLuc3 | TTTTGGCGTCGGTGACCATTTCGAGGGTAGTCAAAATAGATGT |  |  |
| SPluc5 | ATGGTCACCGACGCCAAAAACATAAA |  |  |
| SPluc3 | GTAAGCGGCAGGGTCGGAACAGGAGAG |  |  |
| SPLuc5nest | CTGAGATAGTCGTTGTGCTTGCCATAGA |  |  |
| TSPLuc3nest | AACCGTATTACCGCCTTTGAGTG |  |  |
| ZEBSPLuc5 | ATTGTGGGATCTTGGTCTGT | for FLZEB2 | |
| ZEBSPLuc3 | TTTTTGGCGTCGGTGACCATATTGTCAACCAATGTCATTTA |  |  |
| ZSLuc5nest | AGGGTTAGATGGGTGCTGTGAAT |  |  |
| 657for5 | AGTTATTGTTGCTATGCCCATCTATTC | for Δ*FgLaeA* | |
| 657revtail5 | GCACAGGTACACTTGTTTAGAGCAATTTGGCCTGCGTGTTC |  |  |
| 657fortail3 | CCTTCAATATCATCTTCTGTCGAATTCTTGGCCAGGGTAGTTG |  |  |
| 657rev3 | TGTTTGTCCTGAGCATCTTTCTTTTGTA |  |  |
| 657nest5 | AGGACCCTCAATAGCCACACCCACAG |  |  |
| 657nest3 | TATCATGCACATCGAGAAGACTACAC |  |  |
| Gen-for | CTCTAAACAAGTGTACCTGTGC |  |  |
| Gen-rev | CGACAGAAGATGATATTGAAGG |  |  |
| Gen-forN | TGCTGCTTGGACAAATGAACG |  |  |
| Gen-revN | CCGCTTGGGTGGAGAGGCTATT |  |  |
| Pcrp_for | CTCGAGACAGGACCAGAGAAGC | for OE::*FgLaeA* | |
| Pcrp-GFP tai/rev | CTCGCCCTTGCTCACCATTTTGATTGAAGTTTGGAGGGA |  |  |
| GFP_for | GTGAGCAAGGGCGAGGAGC |  |  |
| GFP_rev | CTTGTACAGCTCGTCCATGCCGTGAG |  |  |
| LaeA-GFP tail | CATGGACGAGCTGTACAAGGCTGTGATGCCTCCACCAAAC |  |  |
| LaeA 3rd Fusion | TTCAGATGGTGCTTATGAGCTTCAT |  |  |
| LaeA rev | GAGCCCCGAAACGAACGAG |  |  |
| LaeA5R | CTTCTGCAGATTTGAAACCGACGA | for *FgLaeA* add-back | |
| LaeA3F | TGCCGTCATATTGGTTTACAGA |  |  |
| PLaeA rev | CTCGCCCTTGCTCAC CATAGAGGAAATTGTGGAAGGTC | for native *FgLaeA*  promoter | |
| pLaeA for | GTTATTGTTGCTTGACTGATTGAT |  |  |
| 657for4 | CGCATTTGAGAAGTGGGCAGAGC | for qRT-PCR | |
| 657rev4 | CAGAAGACCAGGGGCAGACATAGG |  |  |
| EF1-PS1 | GGCTTTCACCGACTACCCTCCTCT |  |  |
| EF1-PS2 | ACTTCTCGACGGCCTTGATGACAC |  |  |
| 9438rev | ATCCTTCTGCACAACACCCTTTAT |  |  |
| 9438for | GACCGATCCTTACGACGACTCTG |  |  |
| 3536for | TTGACAACTTCCCCACATACTCTC |  |  |
| 3536rev | GTGAAGGTGGGAAGGGCGATAAG |  |  |
| 7798for | GAGAATGGTGATGGAGACAGTGCT |  |  |
| 7798rev | TCCAGTTTTCCAGATGCGTTCA |  |  |
| 08081FOR | GTCTCAACCTCAAGGGCGATGCTC |  |  |
| 08081REV | CGTTCCGATGTCCGTGTGC |  |  |
| 11660for | ATCCTGATTCACGGGCCTAACCTC |  |  |
| 11660rev | CGATTCGCCCCAAAAGACAGACAG |  |  |
| MAT1-1/for2 | CTGGAAGAACTGGGCATCGTAA | for qRT-PCR of *MAT1-1-1* | |
| MAT1-1/rev2 | GATATTCTTGTGGCTGGCTACTTT |  |  |
| MAT2/for8 | TGGCAGACGACATTAAGGAGGAGCAC | for qRT-PCR of *MAT1-2-1* | |
| MAT2/rev8 | TGAGCAGCGACAGCAGCAGCAAGAA |  |  |
| VeAcLUC/FOR2 | TCCCGGGGCGGTACCGCGACACCTTCAGCAATTCC | for *FgVeAcLUC* | |
| VeAcLUC/REV2 | TGGATCCCCGGGTACCTTAATACTCGTATTTGTTGAA |  |  |
| LaeAnLuc-for2 | AGCTCGAGTAGTCGACATGGCTGTGATGCCTCCACC | for *FgLaeAnLUC* | |
| LaeA-inrev3 | GCGTACGAGATCTGGTCGACCTGTGGCGGGCCAGGCTTCCT |  |  |
| VIP1-forNT | GAAGCTCGAGTAGTCGACATGGCTGTCGTTGAAGCAGAT | for *FgVIP1nLUC* | |
| VIP1-revNT | TACGAGATCTGGTCGACCGCGTCAGAGCTCTCAGGCTT |  |  |
| LaeAclucfor | GTCCCGGGGCGGTACCGCTGTGATGCCTCCACCAAAC | for *FgLaeAcLUC* | |
| LaeAclucrev | TGGATCCCCGGGTACCTTACTGTGGCGGGCCAGGCTT |  |  |
| Pcrp_rev | CATTTTGATTGAAGTTTGGAGG | for *FgVelBcLUC* |  |
| VELBINpcry/FOR | CAAACTTCAATCAAAATGAACTCGTCCTATCACCCTCCCGAC |  |  |
| VELBNrev/rev | TACGAGATCTGGTCGACGTTCTGATCGTACATCATCTC |  |  |
| nLUCfor | CAGATCTCGTACGCGTCCCGGG |  |  |
| nLUCrev | TCATCCATCCTTGTCAATCAA |  |  |
| nLUCrev2 | ATTAGAGGCCACGATTTGACAC |  |  |

|  |  |
| --- | --- |
